# Validation of candidate gene markers for marker-assisted selection of potato cultivars with improved tuber quality

**DOI:** 10.1007/s00122-012-2035-z

**Published:** 2013-01-09

**Authors:** Li Li, Eckhard Tacke, Hans-Reinhardt Hofferbert, Jens Lübeck, Josef Strahwald, Astrid M. Draffehn, Birgit Walkemeier, Christiane Gebhardt

**Affiliations:** 1 Max Planck Institute for Plant Breeding Research, 50829 Cologne, Germany; 2 BIOPLANT GmbH, 29547 Ebstorf, Germany; 3 Böhm-Nordkartoffel Agrarproduktion OHG, 29574 Ebstorf, Germany; 4 Saka-Pflanzenzucht GbR, 24340 Windeby, Germany; 5 Present Address: State Key Laboratory of Tree Genetics and Breeding, Northeast Forestry University, 150040 Harbin, People’s Republic of China

## Abstract

Tuber yield, starch content, starch yield and chip color are complex traits that are important for industrial uses and food processing of potato. Chip color depends on the quantity of reducing sugars glucose and fructose in the tubers, which are generated by starch degradation. Reducing sugars accumulate when tubers are stored at low temperatures. Early and efficient selection of cultivars with superior yield, starch yield and chip color is hampered by the fact that reliable phenotypic selection requires multiple year and location trials. Application of DNA-based markers early in the breeding cycle, which are diagnostic for superior alleles of genes that control natural variation of tuber quality, will reduce the number of clones to be evaluated in field trials. Association mapping using genes functional in carbohydrate metabolism as markers has discovered alleles of invertases and starch phosphorylases that are associated with tuber quality traits. Here, we report on new DNA variants at loci encoding ADP-glucose pyrophosphorylase and the invertase *Pain*-*1*, which are associated with positive or negative effect with chip color, tuber starch content and starch yield. Marker-assisted selection (MAS) and marker validation were performed in tetraploid breeding populations, using various combinations of 11 allele-specific markers associated with tuber quality traits. To facilitate MAS, user-friendly PCR assays were developed for specific candidate gene alleles. In a multi-parental population of advanced breeding clones, genotypes were selected for having different combinations of five positive and the corresponding negative marker alleles. Genotypes combining five positive marker alleles performed on average better than genotypes with four negative alleles and one positive allele. When tested individually, seven of eight markers showed an effect on at least one quality trait. The direction of effect was as expected. Combinations of two to three marker alleles were identified that significantly improved average chip quality after cold storage and tuber starch content. In F1 progeny of a single-cross combination, MAS with six markers did not give the expected result. Reasons and implications for MAS in potato are discussed.

**Electronic supplementary material:**

The online version of this article (doi:10.1007/s00122-012-2035-z) contains supplementary material, which is available to authorized users.

## Introduction

Potatoes are grown worldwide for food, feed and industrial uses. The tubers are the source of carbohydrates, high quality protein, essential vitamins, minerals and trace elements. Tuber starch is the basis of various industrial products and is used increasingly as substitute for fossil oil in the generation of chemical compounds, for example, bioplastics. Besides starch quality, tuber starch content and starch yield (starch produced per area unit) are important traits for the production of potatoes for industrial uses. A further important quality criterion arises from the requirements of the food processing industry, which produces chips, French fries and other deep fried products from potatoes. This criterion is the tuber content of the reducing sugars fructose and glucose, which determines the culinary quality of chips and French fries (Brown et al. 1990; Hayes and Thill 2002; Kirkman 2007; Mackay et al. 1990; Xiong et al. 2002). Reducing sugars undergo with amino acids at high temperatures a non-enzymatic Maillard reaction, which results, depending on the amount of reducing sugars in the tubers, in unacceptable dark colored products (Talburt et al. 1975). The amount of reducing sugars increases when tubers are stored at temperatures below 10 °C, which are preferred by the industry to inhibit sprouting and to extend marketability. This cold-induced sweetening is an adaptive response to osmotic stress (Sowokinos 2001). In mature dormant tubers, the sugars are produced by degradation of a small fraction of starch (Isherwood 1973). Tuber starch and sugar content are, therefore, connected and part of the same metabolic network.

To meet the demands of farmers, industry and consumers, potato breeding seeks to develop improved varieties, which combine high yield with tuber traits optimized for the various end uses and resistance to pests and diseases. Genetic variation is generated by crossing heterozygous, tetraploid parents and selection is applied in the segregating F1 generation. F1 genotypes are vegetatively propagated and evaluated in multiple year and location trials for some 40 characters, many of them quantitative and modified by the environment (G × E interactions). The selection process from the initial cross to the release of a new variety requires 10–15 years (Milbourne et al. 2007). To assess reliably traits such as tuber yield, starch yield and chip quality, sufficient numbers of tubers are needed, which become available only after several years of vegetative multiplication. Tuber yield and starch content are conveniently evaluated by measuring tuber weight and specific gravity, respectively, which are both non-destructive methods. Chip quality is evaluated by a destructive frying test, by which chip color is rated from light yellow to dark brown. The chip color darkens with increasing amounts of reducing sugars. Due to the potato’s low multiplication rate, the phenotypic selection of high yielding cultivars with good processing quality early in the selection cycle is not reliable. DNA-based markers diagnostic for tuber yield, starch yield and chip quality could circumvent this difficulty and drastically reduce the number of cultivars to be further propagated and evaluated in field trials. Ideally, diagnostic markers are derived from the gene variants (alleles) that are causal for natural variation of a character of interest. The causal variants may be located in the promoter region affecting gene expression, in the coding region affecting protein performance or in other regulatory sequences (introns, 3’ and 5′ untranslated regions). As knowledge of these causal genes and alleles is scarce for most plant agronomic traits, markers that are physically closely linked and, therefore, in linkage disequilibrium with the causal genes can be used as well for marker-assisted selection (MAS).

Tuber yield, starch and sugar content are complex traits controlled by multiple genetic and environmental factors. The prediction of trait values requires, therefore, sets of diagnostic markers, which tag the most important loci controlling the phenotypic variation. Molecular linkage mapping in experimental, mostly diploid populations derived from two parents identified a number of these loci as QTL (quantitative trait locus) (Bonierbale et al. 1993; Douches and Freyre 1994; Freyre and Douches 1994; Menendez et al. 2002; Schäfer-Pregl et al. 1998). QTL mapping and the generation of a molecular function map for carbohydrate metabolism and transport (Chen et al. 2001) provided the basis for adopting a candidate gene approach to identify genes that are causal for natural variation of tuber starch and sugar content. Genes functional in carbohydrate metabolism and transport, including invertases, ADP-glucose pyrophosphorylase, starch phosphorylases, sucrose phosphate synthase and sucrose synthase co-localized with QTL for tuber starch and sugar content (Gebhardt et al. 2005).

In a next step, functional and positional candidate genes were tested for association with tuber yield, starch content, starch yield and chip quality in populations of varieties and advanced breeding clones that were generated in commercial breeding programs. In contrast to the experimental populations used for linkage mapping, this material originated from multiple parental genotypes and represented the common allelic variation present in advanced potato germplasm in Europe. Associations with tuber quality traits were found for DNA polymorphisms at loci encoding invertases, starch phosphorylases, soluble starch synthase I, glucose-6-phosphate dehydrogenase and ribulose bisphosphate carboxylase activase. Individual markers explained up to 12 % of the total variation and, depending on the trait, six to ten markers explained between 26 % (tuber yield) and 55 % (tuber starch content) of the total variation (Li et al. 2005, 2008). Epistatic interactions between candidate gene alleles for tuber starch content and starch yield were also found (Li et al. 2010).

A further step towards MAS is the validation of associated markers by using them to select genotypes with specific marker combinations. The efficiency of the genotypic selection is subsequently assessed by comparative phenotypic evaluation of groups of genotypes with different marker combinations. Markers can also be validated by testing whether the expected phenotypic effects are reproducible in new populations different from the one, in which the marker-trait association was originally identified.

ADP-glucose pyrophosphorylase (*AGPase*) is an important candidate gene that has not been fully evaluated for association with tuber quality traits. AGPase is a key enzyme in starch biosynthesis in higher plants (Tetlow et al. 2004). Potato AGPase, like all higher plant AGPases studied so far, is a heterotetramer composed of two molecules each of two distinct subunits, a large regulatory subunit (LS) and a small catalytic subunit (SS) (Okita et al. 1990). Potato AGPase LS and SS encoding genes (referred to as *AGPaseS* and *AGPaseB*, respectively) have been cloned and characterized (Müller-Röber et al. 1990; Nakata et al. 1991; Okita et al. 1990). Molecular mapping identified three loci for the large subunit *AGPaseS* on potato chromosomes I, IV and VIII, and two for the small subunit *AGPaseB* on chromosomes VII and XII (Chen et al. 2001). The *AGPaseS* loci, in particular, the locus *AGPaseS*-*a* on chromosome I, co-localize with QTL for tuber starch and/or sugar content in experimental mapping populations (Gebhardt et al. 2005). Whereas a minor association with chip quality has been found for an *AGPaseB* allele (Li et al. 2008), *AGPaseS* loci have not been tested for association with tuber quality traits.

*Pain*-*1* on potato chromosome III (Chen et al. 2001) encodes soluble acid invertase. Invertases cleave sucrose into glucose and fructose and are, therefore, the most direct functional candidate genes for chip quality (Sowokinos 2001). Two highly similar *Pain*-*1* cDNA alleles have been identified, which were associated with tuber quality traits. One was more strongly associated with tuber starch content and the other with chip quality after cold storage (Draffehn et al. 2010). DNA variation in the *Pain*-*1* promoter region has not been analyzed for association with tuber quality traits, which might help to further dissect *Pain*-*1* alleles and their phenotypic effects.

In this present study, we report (1) novel associations of *AGPaseS* alleles with tuber quality traits, (2) refined marker-trait associations at the invertase locus *Pain*-*1*, (3) the development of user-friendly polymerase chain reaction (PCR) assays for candidate gene alleles associated with tuber quality traits, (4) the first results of MAS for tuber quality and (5) the validation of previously identified marker-trait associations in new germplasm.

## Materials and methods

### Plant material

A population of 243 tetraploid cultivars (Li et al. 2008) consisting of 34 standard varieties and 90, 96 and 23 breeding clones from Böhm-Nordkartoffel Agrarproduktion OHG (BNA, Ebstorf, Germany), Saka Pflanzenzucht GbR (Windeby, Germany) and Nordring-Kartoffelzucht-und Vermehrungs-GmbH (NORIKA, Groß Lüsewitz, Germany), respectively, was used for association mapping of new candidate genes and development of allele-specific marker assays. This population is referred to as the ‘CHIPS-ALL’ population and has been evaluated in replicated field trials for chip color after harvest (CQA) as well as after cold storage at 4 °C (CQS), for tuber yield (TY), starch content (TSC) and starch yield (TSY) (Li et al. 2008). All traits are correlated with each other. Tuber yield correlates negatively with tuber starch content and chip quality, whereas tuber starch content, starch yield and chip quality are positively correlated (Table 1). MAS and marker validation were performed on two types of material. First, 500 advanced tetraploid ‘BNC’ clones from the breeding program of BNA involving multiple parental lines were used. The BNC clones originated from the 5th to 8th year of phenotypic selection after crossing. Second, 576 ‘SKC’ clones derived from the cross ‘Diana’ × ‘Candella’ were used. The 576 SKC clones were selected in 2007 from 746 seedlings based on general vigor and health. ‘Diana’ was one of the standard varieties in the CHIPS-ALL population, contained most positively associated markers and has relatively good chip quality (CQA = 7.8, CQS = 4.7). Average scores for chip quality of ‘Candella’ are lower (CQA = 6.0, CQS = 3.6). Tuber starch content was 17 % (average from 2002 and 2003) and 18 % (average from 2009 and 2010) for ‘Diana’ and ‘Candella’, respectively.

**Table 1:** Pearson’s correlation between tuber quality traits in the CHIPS-ALL population

Marker	CQS	TSC	TY	TSY
CQA	0.715***	0.586***	−0.310***	0.301***
CQS		0.678***	−0.269***	0.390***
TSC			−0.182**	0.730***
TY				0.528***

** *p* < 0.01, *** *p* < 0.001

### Phenotyping

Chip quality (score of chip color), tuber starch content (% fresh weight), yield (dt/ha) and starch yield (dt/ha) were evaluated as described previously (Li et al. 2008). Chip color was rated from 1 to 9, where 1 corresponded to very dark (very bad) and 9 to very light (very good) chip color. Marker-selected BNC and SKC clones were field propagated in 2009 and 2010 (SKC: Six plants per plot, one replication in 2009, two replications in 2010; BNC: ten plants per plot, two replications each in 2009 and 2010) under standard phytosanitary regimes at Ebstorf (BNA) and Windeby (SaKa), respectively. After harvest, bulked tubers of the BNC clones were evaluated once for tuber starch content (TSC-09, TSC-10), yield (TY-09, TY-10) and starch yield (TSY-09, TSY-10). Chip quality was assessed a first time after harvest (CQA-09, CQA-10), a second time after 2 months storage at 7 °C (CQS7-09, CQS7-10) and a third time after 4 months storage at 5 °C (CQS5-09, CQS5-10). Chip color was obtained from rating ten tubers per clone, time point and year. The trait means over 2 years are referred to as CQA, CQS7, CQS5, TSC, TY and TSY. Due to limited availability of tubers in 2009, the SKC clones were evaluated 2009 in one replication only. As the SKC clones were early in the selection cycle, where yield is not yet stabilized, only tuber starch content was determined in bulked tubers (TSC-09, TSC-1-10, TSC-2-10). Chip quality was scored after harvest (CQA-09, CQA-1-10, CQA-2-10), after 4 months storage at 8 °C (CQS8-09, CQS8-1-10, CQS8-2-10) or 4 °C (CQS4-09, CQS4-1-10, CQS4-2-10). Chip color was obtained from rating three tubers per clone, time point and year. Trait means over the two replications in 2010 are coded as TSC-10, CQA-10, CQS8-10 and CQS4-10. Average chip quality over both years is referred to as CQA, CQS8 and CQS4.

### DNA extraction

Leaf material was harvested from field-grown plants, freeze dried and stored at −20 °C. DNA was extracted from 10 to 30 mg freeze dried leaf tissue per clone in racks with 2 ml safe-lock microcentrifuge tubes arranged in the 96-well format. Two 3 mm tungsten-carbide beads (Qiagen, Hilden, Germany) were added to each tube. Freeze dried leaves were grinded to a fine powder with a Retsch Mixer Mill MM300 (Qiagen, Hilden, Germany). Total genomic DNA was extracted using the BioSprint DNA Plant Kit and a BioSprint workstation (Qiagen, Hilden, Germany). DNA quality and quantity were determined using a spectrophotometer. The quality of the DNA re-isolated in 2009 from BNC and SKC clones selected in 2008 was additionally checked by PCR amplification of a 250 base pair ubiquitin gene fragment, using the primers 5′ GACCATCACTCTTGAGGTTGAG 3′ (forward) and 5′ AATGGTGTCTGAGCTCTCGAC 3′ (reverse) and standard PCR conditions.

### Association mapping of single strand conformation polymorphism (SSCP) markers in the CHIPS-ALL population

Amplicons were generated by PCR from genomic DNA as described (Li et al. 2008), using primers specific for ADP-glucose pyrophosphorylase S (*AGPaseS*, accession X61187) and a 1 kbp promoter fragment of soluble acid invertase (*Pain*-*1*, accession HQ197978). Primer sequences, annealing temperatures and amplicon sizes are specified in Table 2. SSCP analysis of the amplicons was performed as described (Li et al. 2005; Orita et al. 1989). SSCP fragments were scored as present (1) or absent (0) without considering allele dosage. Association analysis with the traits CQA, CQS, TSC, TY and TSY was performed using the model detailed in Li et al. (2008) and the GLM procedure of SPSS 15.0 software (SPSS GmbH, Munich, Germany).

**Table 2:** Locus and marker information for *AGPaseS* and *Pain*-*1*

Locus (chromosome)	Encoded protein	Primer name	Primer sequence (5′–3′)	Transcript size (bp)	PCR product size (bp)	No. of DNA fragments scored (assay type)	*T*_A_ (°C)
*AGPaseS*-*a* (I)	ADP-glucose pyrophosphorylase, large subunit S	AGPsS-7	f-gttcgtagatatgccacgtctt r-acctcgacaaatccatctcc	297	580 + 500	2 (SCAR)	57
		AGPsS-9	f-cagattttgggctggtcaagatt r-tgttccaatgtcttcccaat	311	600	1 (SSCP)	56
		AGPsS-10	f-ccagcagctattgacgattacaa r-atccttcctctggtcggtct	505	1,200	2 (SSCP)	61
*Pain*-*1* (III)	Soluble acid invertase	Pain1_prom_	f-gaccatacgtggctgacaaaattc r-ctggaatggtactgcgtggc	–	1,020	7 (SSCP)	63-58^a^

*SCAR* sequence characterized amplified region, *SSCP* single strand conformation polymorphism

^a^Touch down PCR: The annealing temperature in the initial cycle was set 5 °C above the optimal *T*_A_ of the primers. In subsequent cycles, *T*_A_ was decreased in steps of 1 °C/cycle for five cycles and then maintained for 30 cycles

### Markers used for marker-assisted selection and validation

**Fig. 1:**
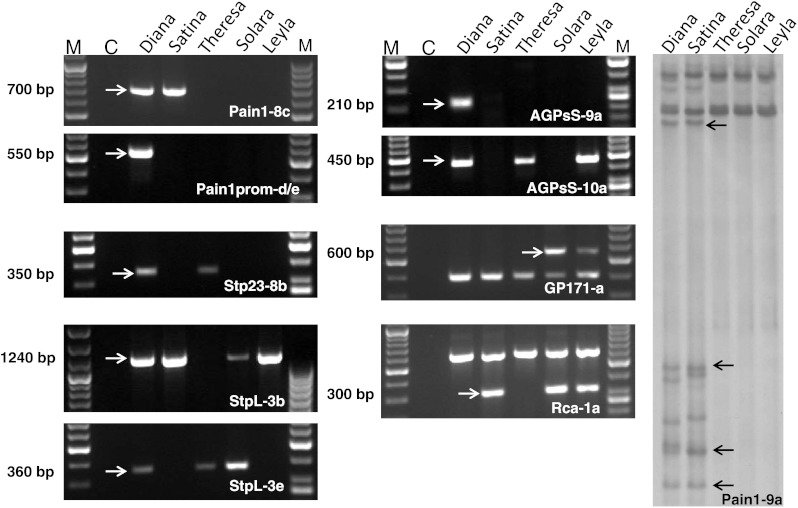
Gel pictures of the markers used for MAS. Amplicons were generated from genomic DNA of the tetraploid varieties Diana, Satina, Theresa, Solara and Leyla. Amplicons of the markers Pain1-8c, Pain_prom_-d/e, Stp32-8b, StpL-3b, StpL-3e, AGPsS-9a, AGPsS-10a, GP171-a and Rca-1a were separated on standard agarose gels and Pain1-9a on SSCP gels. Allele-specific fragments are indicated by* arrows* and their size is shown on the* left* hand side. M: size marker; C: negative PCR control

Markers were chosen based on the most significant associations with tuber quality traits detected in previous association mapping experiments (Li et al. 2005, 2008) (this paper). The markers *GP171*-*a* and *Rca*-*1a* were amplified and scored as direct PCR fragment length polymorphisms on agarose gels as described (Li et al. 2008) (Fig. 1). The GP171 amplicon originated from an anonymous RFLP (restriction fragment length polymorphism) marker (Gebhardt et al. 1991), whilst *Rca*-*1a* corresponds to allele *1a* of ribulose bisphosphate carboxylase activase (RuBisCo activase, *Rca*). The marker allele *InvGE*-*6f* is diagnostic for the group *a* alleles of the apoplastic invertase gene *InvGE* (Draffehn et al. 2010) and was scored as direct PCR fragment length polymorphism as described (Li et al. 2005). The SSCP alleles *Pain1*-*9a* and *Pain1*-*8c* both correspond to the group *a* alleles of the vacuolar acid invertase gene *Pain*-*1*. However, only a subset of genotypes that possesses allele *Pain1*-*9a* also has *Pain1*-*8c,* and the two alleles show slightly different associations. *Pain1*-*9a* is equivalent to the single nucleotide polymorphism (SNP) allele *Pain1*-*A*_*1544*_, whereas *Pain1*-*8c* is equivalent to the SNP alleles *Pain1*-*A*_*718*_ and *Pain1*-*C*_*552*_ (Draffehn et al. 2010). Genotyping for *Pain1*-*9a* was performed by SSCP analysis as described (Li et al. 2008). *Pain1*-*8c* and the new *Pain1*_*prom*_-*d/e* allele were scored using specific PCR assays (see below). The SSCP alleles *Stp23*-*8b, StpL*-*3b* and *StpL*-*3e* originate from two plastidic starch phosphorylase genes (Li et al. 2008). Genotyping for these three alleles as well as for the new *AGPaseS* alleles *AGPsS*-*9a* and *AGPsS*-*10a* was performed using allele-specific PCR assays (see below).

### Development of allele-specific PCR assays

Except for *AGPsS*-*9a*, allele-specific PCR assays were developed as follows: PCR products were generated with the same primers as used for SSCP analysis from two to three genotypes of the CHIPS-ALL population, which either or not carried the targeted SSCP alleles. PCR products were separated on standard agarose gels, excised from the gels, purified with the QiaEx gel-extraction kit (Qiagen, Hilden, Germany) and cloned in the pGEM-T vector system I (Promega, Madison, USA) according to the manufacturer’s instructions. Clones carrying different SSCP alleles present in a heterozygous genotype were identified by SSCP analysis of the PCR products generated with the same primers from recombinant plasmids. For each SSCP allele, 20–30 plasmid insertions were sequenced to obtain a consensus sequence. All sequences were generated at the core facility for DNA analysis of the Max Planck Institute for Plant Breeding Research. The dideoxy chain-termination sequencing method was employed using an ABI PRISM Dye Terminator Cycle Sequencing Ready Reaction Kit and an ABI PRISM 3730 automated DNA Sequencer (Applied Biosystems, Weiterstadt, Germany). DNA sequence analyses were performed using Lasergene software (DNASTAR, Madison, USA). The sequences were aligned to detect SNPs or insertion–deletion polymorphisms specific for the associated SSCP allele, which were then used for primer design (supplementary Figure 1). In some cases, allele-specific primers were designed based on the method ‘PCR Amplification of Specific Alleles’ (PASA) (Okimoto and Dodgson 1996; Sommer et al. 1992). The allele-specific nucleotide was placed at the 3′ end of the primer and one mismatch was introduced at the third nucleotide position from the 3′ terminus. Primers specific for the *AGPsS*-*9a* allele were designed based on two diagnostic SNPs identified by direct amplicon sequencing in the CHIPS-ALL association panel (unpublished data). PCR amplification was performed in 25-μl reaction mixture (20 mM Tris–HCl, pH 8.4, 1.5 mM MgCl_2_, 50 mM KCl) containing 50–60 ng DNA template, 0.4 μM of each primer, 0.2 mM dNTP and 1 U Taq DNA polymerase (Invitrogen, Darmstadt, Germany). Touchdown PCR was used in some cases to increase PCR specificity. The annealing temperature in the initial cycle was set 5 °C above the optimal annealing temperature (*T*_A_) of the primers (Table 4). In subsequent cycles, *T*_A_ was decreased in steps of 1 °C/cycle for five cycles and maintained for 30 cycles. The extension times were adjusted according to the amplicon size as follow: 30 s for products smaller than 500 bp, 45 s for 500–750 bp products and 1 min 30 s for 1,000–1,500 bp products. The amplicons were separated on 2 % agarose gels and visualized by ethidium bromide staining.

### Test statistics for marker validation experiments

All markers were scored as 1 for presence and 0 for absence in a given genotype. Single markers with two character states were tested for significant phenotypic differences between genotype groups (*p* < 0.05) by the *t* test for tuber starch content, yield and starch yield, and Mann–Whitney *U* test for chip quality. Marker combinations were analyzed using analysis of variance (ANOVA) for tuber starch content, yield and starch yield, and Kruskal–Wallis test for chip quality. All analyses were performed with SPSS 15.0 software (SPSS GmbH Software, Munich, Germany).

## Results

### Associations of *AGPaseS* and *Pain*-*1* SSCP markers with tuber quality traits

Three primer pairs were designed in exon sequences of the *AGPaseS* gene (Nakata et al. 1991) (Table 2, supplementary Figure 1). One primer pair (AGPsS-7) generated directly polymorphic PCR products, which did not show association with tuber quality traits. The remaining two primer pairs produced monomorphic PCR products, which yielded three scorable, polymorphic SSCP markers after restriction with *Mse*I. These three markers did show significant (*p* < 0.01) associations with TSC, TSY, CQA and CQS but not with TY (Table 3). The SSCP marker AGPsS-9a was positively associated with all four traits (presence of the marker increased on average tuber starch content, starch yield and chip quality), whilst AGPsS-10a was negatively associated (presence of the marker decreased on average tuber starch content, starch yield and chip quality). The SSCP marker AGPsS-10b showed small, positive associations with CQA, CQS and TSC. Blasting nucleotide sequences of AGPsS-9 and AGPsS-10 amplicons against the potato genome sequence (PGSC 2011) revealed that they were derived from the *AGPaseS*-*a* locus on chromosome I.

**Table 3:** Significant associations between SSCP markers and tuber quality traits in the CHIPS-ALL population

Locus	Chromosome no.	SSCP fragment	Fragment frequency (%)	CQA p (*R* ^2^)^a^	CQS p (*R* ^2^)	TSC p (*R* ^2^)	TSY p (*R* ^2^)
*AGPase*S	I	*AGPsS*-*9a*	19	0.003 ↑^c^ (3.8)	0.000 ↑ (6.9)	0.000 ↑ (8.7)	0.002 ↑ (4.1)
		*AGPsS*-*10a*	41	0.017 ↓ (2.4)	0.009 ↓ (2.9)	0.000 ↓ (11.4)	0.000 ↓ (7,1)
		*AGPsS*-*10b*	54	0.008 ↑ (3.0)	0.044 ↑ (1.8)	0.021 ↑ (2.3)	ns^b^
*Pain*-*1*	III	*Pain1*_*prom*_-*a*	28	0.001 ↑ (4.5)	0.000 ↑ (11.1)	0.000 ↑ (9.5)	0.000 ↑ (5.3)
		*Pain1*_*prom*_-*d/e* ^d^	5	0.000 ↑ (6.0)	0.000 ↑ (10.5)	0.046 ↑ (1.8)	ns
		*Pain1*_*prom*_-*g*	34	0.030 ↑ (2.1)	0.008 ↑ (3.2)	0.021 ↑ (2.4)	ns

^a^The amount of variance (in  %) explained by the marker is given by the R^2^ statistic

^b^Not significant, *p* > 0.05

^c^Direction of effect: ↑ the marker allele has a positive effect on the trait (on average higher tuber starch content and starch yield, lighter chip color), ↓ the marker allele has a negative effect on the trait (on average lower tuber starch content and starch yield, darker chip color)

^d^Two SSCP fragments d and e co-segregated in the CHIPS-ALL population

Three of nine scorable SSCP markers derived from the *Pain*-*1* promoter were associated with TSC, TSY, CQA and CQS (Table 3). The distribution of the Pain1_prom_-a and Pain1-9a SSCP markers in the population CHIPS-ALL was highly similar, indicating that both markers detect the same *Pain*-*1* alleles in homology group a (Draffehn et al. 2010). Accordingly, Pain1_prom_-a showed similar associations as Pain1-9a (Li et al. 2008). The marker Pain1_prom_-d/e was detected only in one quarter of the individuals that carried the Pain1-8c marker. The individuals having Pain1_prom_-d/e had a mean rating of 5.4 [standard deviation (SD) 1.6] for chip color after cold storage—the trait of particular interest for MAS—whereas the individuals carrying Pain1-8c had a mean rating of 4.0 (SD 2.0). The mean ratings of individuals lacking both markers Pain1_prom_-d/e and Pain1-8c were 2.3 (SD 2.1) and 2.1 (SD 2.0), respectively. The marker Pain1_prom_-g with small positive associations with CQA, CQS and TSC represents a new *Pain*-*1* allele.

### Development of specific PCR assays for candidate gene alleles associated with tuber quality traits

To facilitate MAS for tuber quality traits, we converted the associated SSCP markers Stp23-8b, StpL-3b, StpL-3e, Pain1-8c (Li et al. 2008), Pain1_prom_-d/e, AGPsS-9a and AGPsS-10a (this paper) into specific PCR assays as described in the “Materials and methods”. Introns showed more allelic sequence variation than exons. Therefore, polymorphisms in introns were mostly used for the design of primers that generated amplicons in the range of 200–1,200 bp (supplementary Figure 1), an optimal size for separation on standard agarose gels (Fig. 1). Allele-specific primers, annealing temperatures and PCR product sizes are specified in Table 4. PCR protocols were optimized using standard varieties of the CHIPS-ALL population with and without the corresponding SSCP markers (Fig. 1). Specificity of the PCR product for the corresponding SSCP marker was assessed by testing for co-segregation of both marker types in the CHIPS-ALL population. The distributions of the allele-specific PCR markers and the original SSCP markers in the CHIPS-ALL population were nearly identical.

**Table 4:** Allele-specific primers

Marker allele	Primer sequence (5′–3′)	*T*_A_ (°C)	PCR product size (bp)
*Pain1*-*8c*	f-cacataatcaacgtgatgtttaagta r-ggtaatagtaattgcttctaaccg	68-63^a^	703
*Pain1*_*prom*_-*d/e*	f-ctcatcataacttattccatcg r-tggaccacgcataagaaat	63	551
*Stp23*-*8b*	f-cgcatcagaaaaaacctcgg r-acctcctcctgaccatcttt	65-60^a^	348
*StpL*-*3e*	f-ggaccctttgtattttcaggat r-aaagctcttccctgaaagaac	65-60^a^	360
*StpL*-*3b*	f-gaagaagtttctctgttagccac r-cgagtgacgtctgtagttatactagt	65-60^a^	1236
*AGPsS*-*9a*	f-ctgctttcttgcttagttttacc r-catttttcagaaattatatcaggtg	63	210
*AGPsS*-*10a*	f-gaaaatttatcctgaacaaacaccca r-gttaataggaagctaacctcctct	65-60^a^	449

^a^Touch down PCR: the annealing temperature in the initial cycle was set 5 °C above the optimal *T*_A_ of the primers. In subsequent cycles, *T*_A_ was decreased in steps of 1 °C/cycle for five cycles and maintained for 30 cycles

### MAS and marker validation in BNC genotypes

In 2008, five hundred BNC breeding clones from the 5th to 8th year of phenotypic selection were screened for six markers either positively (+) or negatively (−) associated with chip quality, tuber starch content and starch yield: Stp23-8b (+), StpL-3e (+), Pain1-9a (+), AGPsS-10a (−), Rca-1a (−) and GP171-a (−). The Rca-1a marker was not detected in the 500 genotypes. Eleven groups of BNC clones (≥3 individuals per group) were selected based on sharing different combinations of the five remaining markers (Table 5). Group A combined all five positive marker alleles, groups B, C, D, E and F had four positive and one negative marker allele, groups G, J, L had two and groups N and O four negative marker alleles (Table 5). Only one genotype with the ‘all negative’ marker combination was found, which was not sufficient for comparisons of group means and was, therefore, not considered further. In 2009, the marker tests were repeated in the BNC genotypes selected in 2008, using DNA re-extracted from leaves of plants growing 2009 in the field. Seventy-six BNC clones were finally selected that had consistent scores for all markers in both years and were used for subsequent analyses.

**Table 5:** Marker combinations of selected groups of BNC genotypes and their phenotypic means (2-year average)

Genotype group (*n*)^a^	GP171-a	StpL-3e	Stp23-8b	Pain1-9a	AGPsS-10a	CQA (score 1–9) ns	CQS7 (score 1–9)*^c^	CQS5 (score 1–9) ns	TSC (%)***^c^	TY (dt/ha) ns	TSY (dt/ha) ns
A (8)	*0* ^b^	*1*	*1*	*1*	*0*	7.3	6.1	4.1	18.0 a^d^	473.6	85.2
B (5)	**1** ^**b**^	*1*	*1*	*1*	*0*	7.2	5.9	3.6	17.2 a	504.2	85.6
C (3)	*0*	**0**	*1*	*1*	*0*	7.9	6.8	4.9	17.4 a	442.6	77.9
D (8)	*0*	*1*	**0**	*1*	*0*	7.5	7.0	4.5	15.7 b	492.2	77.0
E (8)	*0*	*1*	*1*	**0**	0	6.3	6.4	4.9	16.8 a	534.4	88.7
F (4)	*0*	*1*	*1*	*1*	**1**	6.2	5.5	3.4	17.6 a	450.3	79.2
G (8)	*0*	*1*	**0**	**0**	*0*	7.0	5.2	3.3	15.9 b	511.1	81.5
J (7)	*0*	*1*	*1*	**0**	**1**	8.2	6.2	4.3	16.9 a	488.4	81.6
L (10)	*0*	**0**	**0**	*1*	*0*	7.2	6.6	4.5	16.5 b	508.2	83.0
N (8)	**1**	**0**	**0**	**0**	*0*	6.5	5.3	3.1	14.9 b	519.6	76.8
O (7)	*0*	**0**	**0**	**0**	**1**	5.8	5.6	3.5	14.4 b	486.7	70.2

*ns* not significant

^a^Number of genotypes per group

^b^1: the marker is present, 0: the marker is absent. Bold values indicate an expected negative effect on chip quality, tuber starch content, yield and starch yield, italicized values indicate an expected positive effect

^c^Kruskal–Wallis test (chip quality) or ANOVA (TSC, TY, TSY) for significant differences between groups. * *p* < 0.05, *** *p* < 0.001

^d^Significant differences (LSD) between group A and groups B to O

The 76 selected BNC clones were evaluated in 2009 and 2010 for chip quality after harvest (CQA) and after cold storage (CQS7, CQS5), for tuber starch content (TSC), yield (TY) and starch yield (TSY). Population means and ranges are shown in Table 6. CQA, CQS7 and CQS5 correlated with each other. TSC, TY and TSY were also correlated, TSC negatively with TY. CQA and CQS5 showed positive correlation with TSC (Table 7).

**Table 6:** Descriptive statistics of the traits evaluated in marker-selected BNC and SKC clones

	BNC clones						SKC clones							
	CQA (score)	CQS7 (score)	CQS5 (score)	TSC (%)	TY (dt/ha)	TSY (dt/ha)	CQA-09 (score)	CQA-10 (score)	CQS8-09 (score)	CQS8-10 (score)	CQS4-09 (score)	CQS4-10 (score)	TSC-09 (%)	TSC-10 (%)
Mean	7.0	6.0	4.0	16.4	495.7	80.7	7.6	6.3	5.7	6.9	4.1	3.4	19.0	15.6
SD	1.5	1.3	1.5	1.7	78.8	13.4	0.9	0.9	1.0	0.9	1.0	0.9	1.6	1.7
Minimum	1.0	1.9	1.2	11.6	341.7	49.9	5.0	3.5	3.0	3.7	1.0	1.5	13.7	9.2
Maximum	8.9	8.6	7.4	20.4	702.8	122.9	9.0	8.5	9.0	9.0	7.0	6.5	23.0	19.3

**Table 7:** Pearson’s correlation between traits (2-year means) evaluated in BNC clones

Marker	CQS7	CQS5	TSC	TY	TSY
CQA	0.494***	0.432***	0.325**	−0.371**	ns
CQS7		0.746***	ns	ns	ns
CQS5			0.264*	ns	ns
TSC				−0.240*	0.416***
TY					0.773***

*ns* not significant

* *p* < 0.05, ** *p* < 0.01, *** *p* < 0.001

The trait means over the years 2009 and 2010 of the 11 genotypic groups are included in Table 5. Differences between groups were significant for the traits CQS7 and TSC. Tuber starch content clearly decreased with increasing number of negative marker scores, with the best group A having a 3 % higher average starch content than the worst groups N and O. The same trend was observed for chip quality. Average ratings for chip color were always higher for group A than for groups N and O. The presence of only one or two negative markers in groups B to L did not have an observable effect on chip quality. The absence of the Stp23-8b marker in groups D and G significantly decreased the average tuber starch content when compared with group A.

In addition to the five markers used for MAS, the 76 BNC clones were genotyped with the markers AGPsS-9a (+), Pain1-8c (+) and Pain1_prom_-d/e (+). When tested individually for effects on the 2-year means of the phenotypic traits, seven of the eight markers were significant for one or two traits (Table 8). Results for the single traits in 2009 and 2010 are shown in supplementary Table 1. None of the markers showed significant effects on CQA, TY and TSY. The marker AGPsS-10a had no detectable effect on any trait. The seven significant markers showed the expected positive or negative direction of effect. Of three markers with a positive effect on tuber starch content (Stp23-8b, StpL-3e, Pain1-9a), Stp23-8b was most significant for TSC. Presence of this marker increased tuber starch content on average by 2 % [mean_1_ = 17.3 % (SD 1.7), mean_0_ = 15.5 % (SD 1.3)]. AGPsS-9a was the only marker significant for both traits CQS7 and CQS5 (chip quality after cold storage). Presence of this marker increased the score for chips quality on average by one unit [CQS7: mean_1_ = 6.7 (SD 0.8), mean_0_ = 5.8 (SD 1.3); CQS5: mean_1_ = 4.8 (SD 1.1), mean_0_ = 3.7 (SD 1.5)]. Consistent with previous results (Draffehn et al. 2010), Pain1-9a had a stronger effect on tuber starch content than on chips quality, whereas Pain1-8c and Pain_prom_-d/e, with nearly identical distribution in BNC clones, affected predominantly chips quality [mean_1_ = 6.6 (SD 0.8), mean_0_ = 5.7 (SD 1.4)].

**Table 8:** Significant effects of single markers and marker combinations on tuber quality traits (2-year means) in 76 BNC clones

Marker (no. of genotypes having the marker)	CQS7 (score 1–9)	CQS5 (score 1–9)	TSC (%)
	*p* value^a^	*p* value^a^	*p* value^b^
GP171-a (13)	0.031 ↓^c^	ns	ns
StpL-3e (47)	ns	ns	0.007 ↑
Stp23-8b (35)	ns	ns	<0.001 ↑
Pain1-9a (39)	0.037 ↑	ns	0.003 ↑
Pain1-8c (28)	0.005 ↑	ns	ns
Pain1_prom_-d/e (28)	0.009 ↑	ns	ns
AGPsS-9a (18)	0.004 ↑	0.006 ↑	ns
Pain1-8c/AGPsS-9a	0.001	0.014	ns
Stp23-8b/AGPsS-9a	0.023	0.027	<0.001
Stp23-8b/Pain1-8c	0.004	ns	<0.001
StpL-3e/AGPsS-9a	0.029	0.029	0.047
GP171-a/AGPsS-9a	0.005	0.012	ns
GP171-a/Stp23-8b	ns	ns	<0.001
GP171-a/Pain1-8c	0.009	ns	ns
Stp23-8b/StpL-3e	ns	ns	<0.001
Pain1-8c/AGPsS-9a/Stp23-8b	0.003	0.020	<0.001
GP171/AGPsS-9a/Stp23-8b	ns	ns	<0.001
GP171-a/Pain1-8c/Stp23-8b	0.012	0.041	<0.001

*ns* not significant

^a^Mann–Whitney *U* test or Kruskall-Wallis test

^b^ *t* test or ANOVA

^c^Direction of effect: ↑ the marker has a positive effect on the trait (on average higher tuber starch content and lighter chip color), ↓ the marker has a negative effect on the trait (on average lower tuber starch content and darker chip color)

To identify optimal marker combinations for chip quality and tuber starch content, marker pairs and combinations of three markers were tested for their effect on the traits (Table 8). Seven of 11 marker combinations showed highly significant effects on tuber starch content. All combinations including the marker Stp23-8b increased average tuber starch content (supplementary Table 2). Combinations of the marker AGPsS-9a either with Stp23-8b or Pain1-8c or GP171-a showed significant effects on both CQS7 and CQS5. In agreement with the expectation from previous association studies, the best average scores (5) for chip quality after 4 months storage at 5 °C were obtained when the positive markers AGPsS-9a and Stp23-8b, or AGPsS-9a and StpL-3e were combined, or when AGPsS-9a was present and the negative marker GP171-a was absent. However, when combining AGPsS-9a with Pain1-8c, the genotype class with AGPsS-9a present and Pain1-8c absent scored best (supplementary Table 2). This is contrary to expectation, as Pain1-8c is positively associated with chip quality (Li et al. 2008). This observation was corroborated by the combination of the markers Pain1-8c, AGPsS-9a and Stp23-8b. The highest average scores for CQS5 (5.8) and the highest average tuber starch content (18.2 %) were observed for the genotypic class with AGPsS-9a and Stp23-8b both present but Pain1-8c absent (supplementary Table 2). This indicates that the positive effect of the Pain1-8c marker was converted into the opposite in the presence of the AGPsS-9a marker.

### MAS and marker validation in SKC genotypes

Five hundred and seventy-six F1 genotypes (SKC clones) originating from the cross Diana × Candella were genotyped in 2008 for the segregating markers GP171-a (−), Stp23-8b (+), StpL-3b (−), StpL-3e (+), AGPaseS-10a (−) and InvGE-6f (+). Eighteen groups of SKC clones (≥3 individuals per group) were selected for having in common various combinations of the six markers. Group A consisted of five individuals with all positive markers, whereas three individuals in group P had all negative markers. Ten groups corresponded to five pairs with complementary marker combinations (D1 and D2, F1 and F2, G1 and G2, J1 and J2, K1 and K2) (Table 9). In 2009, the selected SKC clones were propagated in the field, evaluated for chip quality and tuber starch content and re-genotyped with the markers similar to the BNC clones. One hundred and forty-six SKC clones with marker scores consistent with the previous year were finally selected. One hundred and twenty-one SKC clones could be evaluated a second time in 2010 for chip quality and tuber starch content. Unusually dry weather during June and July 2010 lead to a strong reduction in average tuber starch content (Table 6). Marker effects were, therefore, tested separately for 2009 and 2010. Chip quality scores at three different time points and storage temperatures over 2 years were correlated with each other and with TSC-10, whereas TSC-9 was correlated with TSC-10 but not with chip quality (Table 10).

**Table 9:** Marker combinations of selected groups of SKC genotypes and their means for tuber starch content

Genotype group (*n*)^a^	GP171-a	StpL-3b	StpL-3e	Stp23-8b	InvGE-6f	AGPsS-10a	TSC-09 (%)**^c^
A (5)	*0* ^b^	*0*	*1*	*1*	*1*	*0*	18.7 a
B (25)	**1** ^**b**^	*0*	*1*	*1*	*1*	*0*	19.5 a
D1 (6)	*0*	*0*	*1*	**0**	*1*	*0*	20.6 a^d^
D2 (6)	**1**	**1**	**0**	*1*	**0**	**1**	18.4 b
E (8)	**1**	**1**	**0**	*1*	**0**	*0*	17.8 b
F1 (4)	*0*	*0*	*1*	**0**	**0**	*0*	19.8 a
F2 (10)	**1**	**1**	**0**	*1*	*1*	**1**	19.4 a
G1 (3)	*0*	*0*	*1*	*1*	**0**	**1**	17.4 b
G2 (11)	**1**	**1**	**0**	**0**	*1*	*0*	19.1 a
H (3)	**1**	*0*	**0**	**0**	**0**	*0*	16.3 b
J1 (4)	*0*	*0*	**0**	*1*	*1*	**1**	17.4 b
J2 (12)	**1**	**1**	*1*	**0**	**0**	*0*	19.5 a
K1 (3)	*0*	**1**	**0**	**0**	*1*	*0*	18.2 b
K2 (12)	**1**	*0*	*1*	*1*	**0**	**1**	19.0 b
L (5)	0	**1**	*1*	**0**	*1*	**1**	18.0 b
N (3)	**1**	**1**	**0**	**0**	**0**	*0*	19.4 a
O (12)	**1**	**1**	**0**	**0**	*1*	**1**	18.5 b
P (3)	**1**	**1**	**0**	**0**	**0**	**1**	19.6 a

^a^Number of genotypes per group

^b^1: the marker is present, 0: the marker is absent. Bold values indicate an expected negative effect on chip quality, tuber starch content, yield and starch yield, italicized values indicate an expected positive effect

^c^ANOVA for significant differences between groups. ** *p* < 0.01

^d^Significant differences (LSD) between group D1 and the other groups

**Table 10:** Pearson’s correlation between traits (2009 and means 2010) evaluated in SKC clones

Marker	CQA-10	CQS8-09	CQS8-10	CQS4-09	CQS4-10	TSC-09	TSC-10
CQA-09	0.229*	0.216**	0.271**	0.201*	0.303**	ns	0.277**
CQA-10		0.220*	0.610***	0.341***	0.519***	ns	0.358***
CQS8-09			ns	0.229**	0.290**	ns	ns
CQS8-10				0.198*	0.468***	ns	0.359***
CQS4-09					0.440***	ns	0.325***
CQS4-10						ns	0.428***
TSC-09							0.352***

*ns* not significant

* *p* < 0.05, ** *p* < 0.01, *** *p* < 0.001

Except for TSC-09, phenotypic differences between the selected genotypic groups were not significant. The group D1 had the highest tuber starch content in the year 2009 (20.6 %), which differed significantly from other groups, for example, from the complementary group D2 (Table 9). However, unlike the BNC clones, no decrease in tuber starch content with increasing number of negative markers was observed. In fact, the best group A had the same average tuber starch content as the worst group P.

The SKC clones selected based on the six markers described above were genotyped for the additional markers Pain1-8c, Pain1_prom_-d/e and AGPsS-9a. Pain1_prom_-d/e co-segregated with Pain1-8c in the SKC family.

The single markers and combinations of two or three markers were tested for significant effects on chip quality and tuber starch content in 2009 and 2010 (Table 11). When tested individually, the markers GP171-a, Stp23-8b, StpL-3b and InvGE-6f did not show any significant effect, and none of the eight markers and combinations thereof were significant for the chip quality traits CQA-09, CQS8-09 and CQS8-10. Interestingly, the marker StpL-3e showed an effect on tuber starch content in both years, however, with opposite direction, positive as expected in 2009, but negative in 2010. Also AGPsS-10a, which was negatively associated with chip quality in the CHIP-ALL population (Table 3), showed in the SKC clones a small but positive effect on the trait CQS4-09. The positive marker AGPsS-9a showed only a small positive effect on tuber starch content in 2010 (TSC-10), in contrast to the CHIPS-ALL and BNC populations, in which this marker was strongly associated with tuber starch content and chip quality (Tables 3, 8). Consistent with the BNC and CHIPS-ALL populations, the optimal single marker for chip quality was Pain1-8c, which was significant for the 2-year average of chip quality after cold storage at 4 °C (CQS4). Presence of Pain1-8c improved average chip color by 0.4 units. The best pair wise marker combinations for chip quality were Pain1-8c combined with either StpL-3e (Pain1-8c present and StpL-3e absent) or InvGE-6f (both Pain1-8c and InvGE-6f present). The best combinations for tuber starch content were StpL-3e combined with either AGPsS-9a or AGPsS-10a. Also in these cases, the genotypic classes with the highest average tuber starch content differed between the years (supplementary Table 3). Combinations of three markers did not improve the effects on the tuber quality traits compared to pair wise combinations.

**Table 11:** Significant effects of single markers and marker combinations on tuber quality traits in 146 (2009) and 121 (means 2010) SKC genotypes

Marker (no. of genotypes having the marker)	CQA-10 (score 1–9)	CQS4-09 (score 1–9)	CQS4-10 (score 1–9)	CQS4 (score 1–9)	TSC-09 (%)	TSC-10 (%)
	*p* value^a^	*p* value^a^	*p* value^a^	*p* value^a^	*p* value^b^	*p* value^b^
StpL-3e (61-79)	0.043 ↓^c^	ns	ns		0.015 ↑	0.014 ↓
AGPsS-10a (62)	ns	0.014↑	ns		ns	ns
AGPsS-9a (51)	ns	ns	ns		ns	0.022 ↑
Pain1-8c (62-75)	0.038 ↑	0.050 ↑	ns	0.031 ↑	ns	ns
StpL-3e/Pain1-8c	0.010	0.044	ns	ns	ns	0.037
StpL-3e/AGPsS-9a	ns	ns	ns	ns	0.042	0.002
StpL-3e/AGPsS-10a	ns	0.040	ns	ns	0.002	0.002
AGPsS-9a/AGPsS-10a	ns	ns	ns	ns	ns	0.040
Pain1-8c/InvGE-6f	ns	ns	0.041	0.01	ns	ns
InvGE-6f/StpL-3e	0.051	ns	ns	ns	ns	ns

*ns* not significant

^a^Mann–Whitney *U* test or Kruskall-Wallis test

^b^ *t* test or ANOVA

^c^Direction of effect: ↑ the marker has a positive effect on the trait (on average higher tuber starch content and lighter chip color), ↓ the marker has a negative effect on the trait (on average lower tuber starch content and darker chip color)

## Discussion

### Novel markers for tuber quality traits

DNA polymorphisms at the *AGPaseS*-*a* locus on potato chromosome I and in the promoter region of *Pain*-*1* on chromosome III were associated in the CHIPS-ALL population with tuber starch content, starch yield and chip quality before and after cold storage but not with tuber yield (Table 3). The direction of effect of each associated allele was the same for all traits, either positive or negative, meaning that an allele that increased average tuber starch content also increased average chip quality (by decreasing tuber sugar content) and vice versa. These results are consistent with the strong positive correlation between chip quality and tuber starch content as well as starch yield (Table 1). The same relationship is valid for all strongly associated candidate gene alleles identified so far, which function in carbohydrate metabolism (Li et al. 2008). The results of association genetics are in full agreement with the known biochemical links between starch and sugars and the physiology of starch–sugar interconversion (Isherwood 1973). Genes strongly associated exclusively with either tuber starch content or chip quality have not been discovered so far based on the candidate gene approach. Such genes might function in unknown, for example, in regulatory pathways.

Despite the link between tuber starch and sugar content, the relative size of phenotypic effect can vary between alleles of the same locus. Interesting examples are the invertase alleles *Pain1*-*9a, Pain1*-*8c* and the new *Pain1*_*prom*_-*d/e*. *Pain1*-*8c* (= *Pain1*-*A*_*718*_) was in strong LD with *Pain1*-*9a* (= *Pain1*-*A*_*1544*_), but was less frequent in the CHIPS-ALL population and showed stronger association with chip quality than *Pain1*-*9a,* which is predominantly associated with tuber starch content (Draffehn et al. 2010). The genotypes having the allele *Pain1*_*prom*_-*d/e* (12 individuals of the CHIPS-ALL population) were a subset of the genotypes having the *Pain1*-*8c* allele. These genotypes scored on average even better for chip quality after cold storage than genotypes with *Pain1*-*8c*, but showed only a small effect on starch content. The distribution of *Pain1*-*8c* and *Pain1*_*prom*_-*d/e* was nearly identical in the selected BNC population, and the two markers co-segregated in the SKC family, indicating that they are derived from the same haplotype. Alleles with positive trait associations like *Pain1*-*8c*, *Pain1*_*prom*_-*d/e* and *AGPsS*-*9a* had a low frequency in the CHIPS-ALL population. This indicates that there is room for improving processing quality by enriching breeding populations for these low frequency alleles.

### Allele-specific, user-friendly PCR assays for MAS

Most associations with tuber quality traits were discovered based on SSCP analysis (Li et al. 2005, 2008) (this paper). Although this methodology is highly efficient in detecting DNA polymorphisms, it is not suitable for high throughput screening of plant material as required in MAS. We, therefore, converted seven SSCP candidate gene alleles that showed the most promising associations with tuber quality traits in the CHIPS-ALL population into allele-specific PCR assays, which were used for MAS (Table 4; Fig. 1). Primers were designed based on allele-specific SNPs or InDels, and the specificity of the PCR was verified and optimized in individuals of the CHIPS-ALL population. A similar approach was used to develop allele-specific PCR and RT-PCR assays for discrimination of the late blight resistance gene *RB* from numerous *RB* homologs in potato (Millett and Bradeen 2007). Together with the allele-specific SCAR markers InvGE-6f (Li et al. 2005), Rca-1a and GP171-a (Li et al. 2008, 2010), the seven allele-specific, user-friendly markers described in this paper constitute a first set that can be widely used for exploring MAS for tuber quality traits, for analyzing associations in new populations and for allele mining, for example, in landraces or wild potato species.

### Marker validation

MAS for tuber quality traits was exercised in two different types of genetic material. The BNC clones were marker selected from advanced, multi-parental material, which had undergone several years of phenotypic selection, whereas the SKC clones were selected from F1 progeny of a single-cross combination, which has been mildly selected only for general vitality in the first year after crossing. Most markers could be validated in the BNC population, whereas this was largely not the case in the SKC F1 family.

In the 76 BNC genotypes selected based on presence/absence of five allele-specific markers, a clear trend was observed from genotypic groups N and O with the worst allele combinations to group A with the best allele combination. Average tuber starch content, starch yield and chip quality increased from N/O to A as expected (Table 5). The effects of replacing one (groups B–F) or two (groups G, J, L) positive alleles by the complementary negative allele could not be dissected, likely due to the small number of individuals in each genotypic group, which was insufficient to detect small phenotypic effects. An exception was the positive marker Stp23-8b. All five groups (C, G, L, N, O) lacking this marker had a significantly lower tuber starch content compared to the six other groups having it. The strong positive effect of Stp23-8b on tuber starch content was also evident in single marker tests and in combinations with one and two other markers (Table 8). With the exception of AGPsS-10a, all markers tested individually in the 76 BNC genotypes showed a significant effect on at least one tuber quality trait. Except for Stp23-8b, significance levels were lower than in the CHIPS-ALL population, likely due to the small population size. Lack of significant effect, for example, on CQA, may be due to limited phenotypic range. Correlations between traits were weaker due to the small number of phenotyped BNC clones but still consistent with the trait correlations observed in the CHIPS-ALL population. The directions of effects were also consistent with the original associations in the CHIPS-ALL population. A remarkable exception was combination of the markers Pain1-8c and AGPsS-9a, which individually showed reproducible, positive effects on chip quality. However, the genotype group with Pain1-8c absent and AGPsS-9a present scored best for chip quality, particularly after cold storage (supplementary Table 2), suggesting incompatibility between certain alleles of soluble acid invertase and ADP-glucose pyrophosphorylase S. The plastidic starch phosphorylase loci *Stp23* and *StpL* are identical to *PHO1A* and *PHO1B*, respectively, which have been tested for association with tuber starch content in a population of 205 varieties and breeding clones different from the CHIPS-ALL population. SSCP markers at both loci were associated with tuber starch content (Urbany et al. 2011). Together with the results reported here for 76 BNC clones, associations with tuber starch content of allelic variants at these two starch phosphorylase loci have now been validated in three different populations.

The 146 SKC genotypes were selected based on presence/absence of six markers, four of which were the same as used for selecting the BNC genotypes. Significant differences between the 18 genotypic groups were only observed for tuber starch content in 2009, and these differences did not correspond to the expectation of increasing starch content with increasing number of positive markers (Table 9). No differences were detected for chip quality. When tested individually, four of the eight markers, for which the SKC family was genotyped, showed an effect on at least one trait. However, the direction of effect of two markers (StpL-3e, AGPsS-10a) was inconsistent between years and the original associations in the CHIPS-ALL population (Table 11).

One reason for the limited success of MAS in the SKC family could be the phenotypic evaluation, which suffered from two handicaps, the low number of tubers available for testing chip quality in 2009 and highly unusual weather conditions during the growing season 2010. Like tuber yield, the assessment of chip quality in the early years of multiplication might not be reliable enough to validate marker-trait associations that were identified in a population of varieties and more advanced breeding clones. Furthermore, marker-trait associations identified under normal climatic conditions might be unstable under exceptional weather circumstances (G × E interactions). In this respect, Pain1-8c was the only marker that showed a consistent positive effect on chip quality in both BNC and SKC genotypes.

Another reason may be that the markers explain only part of the phenotypic variation of polygenic traits. In this case, the markers can predict the phenotype of an individual only with a certain probability but not with certainty. The prediction of marker effect is probabilistic, not deterministic. The prediction might fail, therefore, in individual cross combinations due to additional, unknown genetic factors and epistatic interactions (Li et al. 2010), which segregate in a particular F1 family. Association mapping with genome wide SNP markers, which now becomes feasible based on the draft potato genome sequence (PGSC 2011), is the strategy to fill the knowledge gaps on how many and which loci control the natural variation of complex tuber traits.

## Conclusion

Marker-assisted selection in applied potato breeding programs is so far restricted to few genes for pathogen resistance with major effects (Ortega and Lopez-Vizcon 2012; Rizza et al. 2006; Whitworth et al. 2009). The implementation of MAS for polygenic traits poses a major challenge. In this paper, we report the results of the first experiments to implement MAS for polygenic tuber quality traits in potato. From this exercise, the following lessons can be learned:

1. Incompatibilities between alleles do occur and have to be taken into account. More important than the number of markers is the choice of suitable marker combinations, for example, the combination of marker Pain1-8c absent with markers AGPsS-9a and Stp23-8b present, which was optimal for improving tuber quality in the BNC clones. In other breeding populations, other marker combinations might perform better.
2. With the current state of knowledge, the most reproducible single marker for increasing average tuber starch content and eventually starch yield and chip quality is Stp23-8b, for improving average chip quality after cold storage Pain1-8c or Pain1_prom_-d/e.
3. MAS for tuber quality should not rely on single-cross combinations but should be applied to multiple parents and their progeny. For example, MAS can first be used to purify parental populations from negative alleles and to increase frequency and dosage of positive alleles. Pre-selection for general plant performance such as vigor will reduce the number of progeny clones to be subjected to MAS (Ortega and Lopez-Vizcon 2012). Phenotypic evaluation of tuber quality can then be performed on the remaining clones at a later stage in the breeding cycle.

## Electronic supplementary material

Below is the link to the electronic supplementary material.

Supplementary material 1 (DOCX 35 kb)

Supplementary material 2 (DOCX 15 kb)

Supplementary material 3 (XLSX 14 kb)

Supplementary material 4 (XLSX 13 kb)
