# Supplementary material for: Validation of candidate gene markers for marker-assisted selection of potato cultivars with improved tuber quality
Source: Theor Appl Genet. 2013 Jan 9;126(4):1039–52. doi: 10.1007/s00122-012-2035-z (PMC3607734; doi:10.1007/s00122-012-2035-z)
Supplement: Supplementary file 1 — Supplementary material 1 (DOCX 35 kb) [file 122_2012_2035_MOESM1_ESM.docx]

***AGPaseS***

*AGPaseS-10a* CCAGCAGCTATTGACGATTACAATGCCCAAGTAAAGGATCTTAGATCTTTCGATTGAAATTATTTTTATGAATTGAGTAC

*AGPaseS-10b* CCAGCAGCTATTGACGATTACAATGTCCAAGTAAAGGATCTTAGATCTTTCGATTGAAATTATTTTTATGAATTGAGTAC

*AGPaseS-10a* ATTAGTTACTCAACTA~~~~~~~~GCTGTAGTTTTAACAAATTTATGCTATCTTCTCAGGCATACATTTTCAAAGACTAT

*AGPaseS-10b* ATTAGTTACTCAACTAAAAAATTAGCTGTAGTTTTAACAAATTTATGCTATCTTCTCAGGCATACATTTTCAAAGACTAT

*AGPaseS-10a* TGGGAGGACATTGGAACAATTAAATCTTTTTATAATGCTAGCTTGGCACTGACACAAGAGGCATGTTGCAAATTAATCTT

*AGPaseS-10b* TGGGAGGACATTGGAACAATTAAATCTTTTTATAATGCTAGCTTGGCACTCACACAAGAGGCATGTTGCAAATTAATCTT

*AGPaseS-10a* TATACTTGTCACCATATGCAATGAACGAACTAATTTTGACTAGGTCAGAAATTATTTTACATCAGTGCATAATCCAACTG

*AGPaseS-10b* TATACTTGTCACCATATGCAATGAAC~~~~TAATTTTGACTATCTCAGAAATTCTTTTACATCAGTGCATAATCCAACTG

*AGPaseS-10a* ATTAATTTCCATTGCAGTTTCCAGAGTTCCAATTTTACGATCCAAAAACACCTTTTTACACATCTCCTAGGTTCCTTCCA

*AGPaseS-10b* ATTAATTTCCATTGCAGTTTCCAGAGTTCCAATTTTACGATCCAAAAACACCTTTT~ACACATCTCCTAGGTTCCTTCCA

*AGPaseS-10a* CCAACCAAGATAGACAATTGCAAGGTAAGTGGGTAAATTGTGCAAGTGTTTTTTTTTTTCATTAACTGGGGTGGGTTCTA

*AGPaseS-10b* CCAACCAAGATAGACAATTGCAAGGTAAGTGGGTAAATTGTGCAAGTGTTTT~~~~~~GCATTAACTGGGG~~~~TTCTA

*AGPaseS-10a* ATAGG~TTTTGTATTCTCTGCTTTTCAGATTAAGGATGCCATAATCTCTCATGGATGTTTCTTGCGAGATTGTTCTGTGG

*AGPaseS-10b* ATAGGGTTTTGTATTCTCTGCTTTTCAGATTAAGGATGCCATAATCTCTCATGGATGTTTCTTGCGAGATTGTTCTGTGG

*AGPaseS-10a* AACACTCCATAGTGGGTGAAAGATCGCGCTTAGATTGTGGTGTTGAACTGAAGGTTTGCTTGTAGCCCTAATTTGAAAAT

*AGPaseS-10b* AACACTCCATAGTGGGTGAAAGATCGCGCTTAGATTGTGGTGTTGAACTGAAGGTTTGCTTGTAGCCCTAATTTGAAAAT

*AGPaseS-10a* TTATCCTGAACAAACACACATTTATTTTCTTTTCTTTAGTTTGTGAAAAATGCACTAGTTTATTATTGTTTCTAGTACAT

*AGPaseS-10b* TTATCCTGAACAAACACA~~TTTATTTTCTTTTCTTTAGTTTGTGAAAAATGCACTAGTTTATT~~~GTTTTTAGTACAT

*AGPaseS-10a* TTTGTTGTTAAGTATAGATCGTTTTACAACCACAGGATACTTTCATGATGGGAGCAGACTTGTACCAAACAGAATCTGAG

*AGPaseS-10b* TTTGTTGTAAAGTATAGA~CGTTTTACAACCACAGGACACTTTCATGATGGGAGCAGACTTGTACCAAACAGAATCTGAG

*AGPaseS-10a* ATTGCCTCCCTGTTAGCAGAGGGGAAAGTACCGATTGGAATTGGGGAAAATACAAAAATAAGGTAAATGGACATTGAATA

*AGPaseS-10b* ATTGCCTCCCTGTTAGCAGAGGGGAAAGTACCGATTGGAATTGGGGAAAACACAAAAATAAGGTAAATGGACATTGAATA

*AGPaseS-10a* TTTCATGAAAATGACATCATTCATACTTG~~~~~~~~~~~~~~~AGAATTCTTTAATTTGATGATTATGGTTTCACTCGT

*AGPaseS-10b* TTTGACACGAAACTCAAAAAAATCTTTTTTGACAAATGTATTTCAGAATTCTTTAATTTGAT~~~TATGGTTTCACTCGT

*AGPaseS-10a* ACAGGAAATGTATCATTGACAAGAACGCAAAGATAGGAAAGAATGTTTCAATCATAAATAAACATGTAAGCTTCTTTAGT

*AGPaseS-10b* ACAGGAAATGTATCATTGACAAGAACGCAAAGATAGGAAAGAATGTTTCAATCATAAATAAACATGTAAGCTTCTTTAGT

*AGPaseS-10a* GTGTCTTATTTTTCAACTATAGTAGAATATTATAG~~~~~~~~~AGCGGAGGTTAGCTTCCTATTAACTATTGTT~~~~~

*AGPaseS-10b* GTGTCTTATTTTTCAACTATAGTAGAATATTATAGTATTTATAGAGCGGAGGTTAGCTTCCTAACAACTATTGTTCACAC

*AGPaseS-10a* ~~~~~~~~~CAAAACACAATAGAGCACGCAACACACATTGTTGCTCGTGAGAAAGAAACCAGTACAGTGTTCAATTCAGA

*AGPaseS-10b* AGTCAAGTTCAAAACACAATAGAGCACACAACACACATTGTTGCTCGTGAGAAAGAAACCAGTACAGTGTTCAATTCAGA

*AGPaseS-10a* TTTGATTGTTTGTAGCATGAGAGCATA~~GAAAACTCTATTGACAAGATCCCACTCGAAAAAAAGAAAACTGTTGCAAGC

*AGPaseS-10b* TTTGATTGTTTGTAGCATGAGAGCATAATACCTAAGAAAAAACTATAGGGACTCTATTGACAAGGAAAACTGTTGCAAGC

*AGPaseS-10a* TATAGAGCACTTATATTTTTGAGACATATATAATTGATTATTAGAATTAACTCT~~~~~~~~CTTTTCTTTGAAACTTTA

*AGPaseS-10b* TATAGAGCAC~~~~~~~~TTGAGACATA~~~~ATTGATTATTAGAATTAACTCTCTGATGTTCTTTTCTTTGAAACTTTA

*AGPaseS-10a* GGGTGTTCAAGAGGCAGACCGACCAGAGGAAGGAT

*AGPaseS-10b* GGGTGTTCAAGAGGCAGACCGACCAGAGGAAGGAT

***Stp23***

*Stp23-8a* GCAACAGCTCAAAGTGTTCGTGATTCGCTCCTTATTAATTGGAATGCTACGTATGATATTTATGAAAAGCTGAACATGAA

*Stp23-8b* GCAACAGCTCAAAGTGTTCGTGATTCGCTCCTTATTAATTGGAATGCTACGTATGATATTTATGAAAAGCTGAACATGAA

*Stp23-8c* GCAACAGCTCAAAGTGTTCGTGATTCGCTCCTTATTAATTGGAATGCTACGTATGATATTTATGAAAAGCTGAACATGAA

*Stp23-8d* GCAACAGCTCAAAGTGTTCGTGATTCGCTCCTTATTAATTGGAATGCTACGTATGATATTTATGAAAAGCTGAACATGAA

*Stp23-8e* GCAACAGCTCAAAGTGTTCGTGATTCGCTCCTTATTAATTGGAATGCTACGTATGATATTTATGAAAAGCTGAACATGAA

*Stp23-8f* GCAACAGCTCAAAGTGTTCGTGATTCGCTCCTTATTAATTGGAATGCTACGTATGATATTTATGAAAAGCTGAACATGAA

*Stp23-8a* GCAAGCGTACTATCTATCCATGGAATTTCTGCAGGTATCTCATTATTCTTACTTTCTCTTTTGCTCTTTTGTATGACTGT

*Stp23-8b* GCAAGCGTACTATCTATCCATGGAATTTCTGCAGGTATCTCATTATTCTTACTTTCTCTTTTGCTCTTTTGTATGACTGT

*Stp23-8c* GCAAGCGTACTATCTATCCATGGAATTTCTGCAGGTATCTCATTATTCTTACTTTCTCTTTTGCTCTTTTGTATGACTGT

*Stp23-8d* GCAAGCGTACTATCTATCCATGGAATTTCTGCAGGTATCTCATTATTCTTACCTTCTCTTTTGCTCTTTTGTATGACTGT

*Stp23-8e* GCAAGCGTACTATCTATCCATGGAATTTCTGCAGGTATCTCATTATTCTTACTTTCTCTTTTGCTCTTTTGTATGACTGT

*Stp23-8f* GCAAGCGTACTATCTATCCATGGAATTTCTGCAGGTATCTCATTATTCTTACTTTCTCTTTTGCTCTTTTGTATGACTGT

*Stp23-8a* GCAGAGTGACCTTAAATTATATCTAGTAAGAAATTAATCCGTTTGATATTTGCTGACAAATTAGACTGTATATTTACTGT

*Stp23-8b* GCAGAGTGACCTTAAATTATATCTAGTAAGAAATTAATCCGTTTGATATTTGCTGACAAATTAGACTGTATATTTACTGT

*Stp23-8c* GCAGAGTGACCTTAAATTATATCTAGTAAGAAATTAATCCGTTTGATATTTGCTGACAAATTATACTGTATATTTACTGT

*Stp23-8d* GCAGAGTGACCTTAAATTATATCTAGTAAGAAATTAATCCGTTTGATATTTGCTGACAAATTAGACTGTATATTTACTGT

*Stp23-8e* GCAGAGTGACCTTAAATTATATCTAGTAAGAAATTAATCCGTTTGATATTTGCTGACAAATTAGACTGTATATTTACTGT

*Stp23-8f* GCAGAGTGACCTTAAATTATATCTAGTAAGAAATTAATCCGTTTGATATTTGCTGACAAATTATACTGTATATTTACTGT

*Stp23-8a* TACACATGAGAGTTTCTGAAATTTATGCACAAACAACTCTTTGCGAGCTGAGTTTAATGTATGCCCTATCTACTGTTTAG

*Stp23-8b* TACACATGAGAGTTTCTGAAATTTATGCACAAACAACTCTTTGCGAGCTGAGTTTAATGTATGCCCTATCTACTGTTTAG

*Stp23-8c* TACACATGAGAGTTTCTGAAATTTATGCACAAACAACTCTTTGCGAGCTGAGTTTAATTTATGCCCTATCTACTGTTTAG

*Stp23-8d* TACACATGAGAGTTTCTGAAATTTATGCACAAACAACTCTTTGCGAGCTGAGTTTAATTTATGCCCTATCTACTGTTTAG

*Stp23-8e* TACACATGAGAGTTTCTGAAATTTATGCACAAACAACTCTTTGCGAGCTGAGTTTAATTTATGCCCTATCTACTGTTTAG

*Stp23-8f* TACACATGAGAGTTTCTGAAATTTATGCACAAACAACTCTTTGCGAGCTGAGTTTAATTTATGCCCTATCTACTGTTTAG

*Stp23-8a* GGTAGAGCATTGTTAAATGCAATTGGTAATCTGGAGCTTACTGGTGCATTTGCGGAAGCTTTGAAAAACCTTGGTCACAA

*Stp23-8b* GGTAGAGCATTGTTAAATGCAATTGGTAATCTGGAGCTTACTGGTGCATTTGCGGAAGCTTTGAAAAACCTTGGCCACAA

*Stp23-8c* GGTAGAGCATTGTTAAATGCAATTGGTAATCTGGAGCTTACTGGTGCATTTGCGGAAGCTTTGAAAAACCTTGGCCACAA

*Stp23-8d* GGTAGAGCATTGTTAAATGCAATTGGTAATCTGGAGCTTACTGGTGCATTTGCGGAAGCTTTGAAAAACCTTGGCCACAA

*Stp23-8e* GGTAGAGCATTGTTAAATGCAATTGGTAATCTGGAGCTTACTGGTGCATTTGCGGAAGCTTTGAAAAACCTTGGCCACAA

*Stp23-8f* GGTAGAGCATTGTTAAATGCAATTGGTAATCTGGAGCTTACTGGTGCATTTGCGGAAGCTTTGAAAAACCTTGGCCACAA

*Stp23-8a* TCTAGAAAATGTGGCTTCTCAGGTCAGTGTGACTTTTATTTCACGCATCAGAAAAAACCTAGTTTTCCAAGTTCTTCTGT

*Stp23-8b* TCTAGAAAATGTGGCTTCTCAGGTCAGTGTGACTTTTATTTCACGCATCAGAAAAAACCTAGGTTTCCAAGTTCTTCTGT

*Stp23-8c* TCTCGAAAATGTGGCTTCTCAGGTCAGTGTGACTTTTATTTCACGCATCAGAAAAAACGTAGTTTTCCAAGTTCTTCTGT

*Stp23-8d* TCTAGAAAATGTGGCTTCTCAGGTCAGTGTGACTTTTATTTCACGCATCAGAAAAAACCTAGTTTTCCAAGTTCTTCTGT

*Stp23-8e* TCTAGAAAATGTGGCTTCTCAGGTCAGTGTGACTTTTATTTCACGCATCAGAAAAAACCTAGTTTTCCAAGTTCTTCTGT

*Stp23-8f* TCTAGAAAATGTGGCTTCTCAGGTCAGTGTGACTTTTATTTCACGCATCAGAAAAAACCTAGTTTTCCAAGTTCTTCTGT

*Stp23-8a* TTTTTACTCAACAACAGTTGGACTATGTGTGAATATTTGTGACTTTGTGCATCGACTGTTATAATATTCAGAAGCCATAC

*Stp23-8b* GTTTTACTCAACAACAGTTGGACTATGTGTATTCATTTGTGACTTTGTGCATCGACTGTTATAACATTCAGAAGCCATAC

*Stp23-8c* GTTTTACTCAACAACAGTTGGACTATGTGTATTCATTTGTGACTTTGTGCATTGACTGTTATAATATTCAGAAGCCATAC

*Stp23-8d* GTTTTACTCAACAACAGTTGGACTATGTGTATTCATTTGTGACTTTGTGCATCGACTGTTATAACATTCAGAAGCCATAC

*Stp23-8e* GTTTTACCCAACAACAGTTGGACTATGTGTATTCATTTGTGACTTTGTGCATCGACTGTTATAATATTCAGAAGCCATAC

*Stp23-8f* GTTTTACTCAACAACAGTTGGACTATGTGTATTCATTTGTGACTTTGTGCATCGACTGTCATAATATTCAGAAGCCATAC

*Stp23-8a* ATGCCATACTTGTTGTGTCTTTATGTTTTTATTTGGAGTTGACTACTTGTTAATTTTTGAATAATTATTGACAGGAGCCA

*Stp23-8b* ATGCCATACTTGTTGTGTCTTTATGTTTTTATTTGGAGTTGACTACTTGTTAATTTTTGAATAATTATTGACAGGAGCCA

*Stp23-8c* ATGCCATACTTGTTGCGTCTTTATGTTTTTATTTGGAGTTGACTACTTGTTAATTTTTGAATAATTATTGACAGGAGCCA

*Stp23-8d* ATGCCATACTTGTTGTGTCTTTATGTTTTTATTTGGAGTTGACTACTTGTTAATTTTTGAATAATTATTGACAGGAGCCA

*Stp23-8e* ATGCCATACCTGTTGTGTCTTTATGTTTTTATTTGGAGTTGACTACTTGTTAATTTTTGAATAATTATTGACAGGAACCA

*Stp23-8f* ATGCCATACTTGTTGTGTCTTTATGTTTTTATTTGGAGTTGACTACTTGTTAATTTTTGAATAATTATTGACAGGAGCCA

*Stp23-8a* GATGCTGCTCTTGGAAATGGGGGTTTGGGACGGCTTGCTTCCTGTTTTCTGGACTCTTTGGCAACACTAAACTACCCAGC

*Stp23-8b* GATGCTGCTCTTGGAAATGGGGGTTTGGGACGGCTTGCTTCCTGTTTTCTGGACTCTTTGGCAACACTAAACTACCCAGC

*Stp23-8c* GATGCTGCTCTTGGAAATGGGGGTTTGGGACGGCTTGCTTCCTGTTTTCTGGACTCTTTGGCAACACTAAACTACCCAGC

*Stp23-8d* GATGCTGCTCTTGGAAATGGGGGTTTGGGACGGCTTGCTTCCTGTTTTCTGGACTCTTTGGCAACACTAAACTACCCAGC

*Stp23-8e* GATGCTGCTCTTGGAAATGGGGGTTTGGGACGGCTTGCTTCCTGTTTTCTGGACTCTTTGGCAACACTAAACTACCCAGC

*Stp23-8f* GATGCTGCTCTTGGAAATGGGGGTTTGGGACGGCTTGCTTCCTGTTTTCTGGACTCTTTGGCAACACTAAACTACCCAGC

*Stp23-8a* ATGGGGCTATGGACTTAGGTACAAGTATGGTTTATTTAAGCAACGGATTACAAAAGATGGTCAGGAGGAGGTG

*Stp23-8b* ATGGGGCTATGGACTTAGGTACAAGTATGGTTTATTTAAGCAACGGATTACAAAAGATGGTCAGGAGGAGGTG

*Stp23-8c* ATGGGGCTATGGACTTAGGTACAAGTATGGTTTATTTAAGCAACGGATTACAAAAGATGGTCAGGAGGAGGTG

*Stp23-8d* ATGGGGCTATGGACTTAGGTACAAGTATGGTTTATTTAAGCAACGGATTACAAAAGATGGTCAGGAGGAGGTG

*Stp23-8e* ATGGGGCTATGGACTTAGGTACAAGTATGGTTTATTTAAGCAACGGATTACAAAAGATGGTCAGGAGGAGGTG

*Stp23-8f* ATGGGGCTATGGACTTAGGTACAAGTATGGTTTATTTAAGCAACGGATTACAAAAGATGGTCAGGAGGAGGTG

***Pain-1***

*Pain1-8c* GCCGTCAAGAGGTGTTTCTCAGGGAGTCTCCGATAAGACTTTTCGAGATGTCGTCAATGCTAGTCACGTTTCTTATGCGT

*Pain1-8a* GCCGTCAAGAGGTGTTTCTCAGGGAGTCTCCGATAAGACTTTTCGAGATGTCGTCAATGCTAGTCACGTTTCTTATGCGT

*Pain1-8b* GCCGTCAAGAGGTGTTTCTCAGGGAGTCTCCGATAAGACTTTTCGAGATGTCGTCAATGCTAGTCACGTTTCTTATGCGT

*Pain1-8d* GCCGTCAAGAGGTGTTTCTCAGGGAGTCTCCGATAAGACTTTTCGAGATGTCGTCAATGCTAGTCACGTTTCTTATGCGT

*Pain1-8c* GGTCCAATGCTATGCTTAGCTGGCAAAGAACTGCTTACCATTTTCAACCTCAAAAAAATTGGATGAACGGTAATTAACTT

*Pain1-8a* GGTCCAATGCTATGCTTAGCTGGCAAAGAACTGCTTACCATTTTCAACCTCAAAAAAATTGGATGAACGGTAATTAACTT

*Pain1-8b* GGTCCAATGCTATGCTTAGCTGGCAAAGAACTGCTTACCATTTTCAACCTCAAAAAAATTGGATGAACGGTAATTAACTT

*Pain1-8d* GGTCCAATGCTATGCTTAGCTGGCAAAGAACTGCTTACCATTTTCAACCTCAAAAAAATTGGATGAACGGTAATTAACTT

*Pain1-8c* TCTTATTTTGACTTTTCTGTAATTTCCTATTTATTTGATCTTAAAATTGAAATAAATTATAAATACTTATACTAGTTTTT

*Pain1-8a* TCTTATTTTGACTTTTCTGTAATTTCCTATTTATTTGATCTTAAAATTGAAATAAATTATAAATACTTATACTAGTTTTT

*Pain1-8b* TCTTATTTTGACTTTTCTGTAATTTCCTATTTATTTGATCTTAAAATTGAAATAAATTATAAATACTTATACTAGTTTTT

*Pain1-8d* TCTTATTTTGACTTTTCTGTAATTTCCTATTTATTTGATCTTAAAATTGAAATAAATTATAAATACTTATACTAGTTTTT

*Pain1-8c* TTTTCTTAATGATATTTATGGCTATTGATCTGTTGGGGTATCTTTTGGATTCTGATTGGATGCTATTCTGCAGATCCTAA

*Pain1-8a* TTTTCTTAATGATATTTATGGCTATTGATCTGTTGGGGTATCTTTTGGATTCTGATTGGATGCTATTCTGCAGATCCTAA

*Pain1-8b* TTTTCTTAATGATATTTATGGCTATTGATCTGTT~GGGTATCTTTTGGATCCTGATTGGATGCTATTCTGCAGATCCTAA

*Pain1-8d* TTTTCTTAATGATATTTATGGCTATTGATCTGTTGGGGTATCTTTTGGATCCTGATTGGATGCTATTCTGCAGATCCTAA

*Pain1-8c* TGGTGAGTTCAAAGTTAATTATCATCACTATTTTCTGTTAGTATTTAATTAATTATATTCTTAAACCATGAATTAAAACT

*Pain1-8a* TGGTGAGTTCAAAGTTAATTATTATCACTATTTTCTGTTAGTATTTAATTAATTATATTCTTAAACCATGAATTAAAACT

*Pain1-8b* TGGTGAGTTCAAAGTTAATTATCATCACTATTTTCTGTTAGTATTTAATTAATTATATTCTTAAACCATGAATTAAAACT

*Pain1-8d* TGGTGAGTTCAAAGTTAATTATCATCACTATTTTCTGTTAGTATTTAATTAATTATATTCTTAAACCATGAATTAAAACT

*Pain1-8c* TTAAAGGCAGTAAAATTCTCTCATGAGGTAATTATGGTTTTATTTGATTTAAGCCTATAAGTGCCAACCAATCCATGTAT

*Pain1-8a* TTAAAGGCAGTAAAATTCTCTCATGAGGTAATTATGGTTTTATTTGATTTAAGCCTATAAGTGCCAACCAATCCATGTAT

*Pain1-8b* TTAAAGGCAGTAAAATTCTCTCATGAGGTAATTATGGTTTTATTTGATTTAAGCCTATAAGTGCCAACCAATCCATGTAT

*Pain1-8d* TTAAAGGCAGTAAAATTCTCTCATGAGGTAATTATGGTTTTATTTGATTTAAGCCTATAAGTGCCAACCAATCCATGTAT

*Pain1-8c* GAGCAAATCATTAATTCGGGTGCTTATGTATGTCATCTCGGTTAATCCTTTTACCTTTTACTCAAAAGGAACTATTACTC

*Pain1-8a* GAGCAAATCATTAATTCGGGTGCTTATGTATGTCATCTCGGTTAATCCTTTTACCTTTTACTCAAAAGGAACTATTACTC

*Pain1-8b*  GAGCAAATCATTAATTCGGGTGCTTATGTATGTCATCTCGATTAATCCTTTGACCTTTTACTCAAAAGGAACTATTACTC

*Pain1-8d* GAGCAAATCATTAATTCGGGTGCTTATGTATGTCATCTCGGTTAATCCTTTTACCTTTTACTCAAAAGGAACTATTACTC

*Pain1-8c* CGTCCAAAATAATTGATGTTTCACATAATCAACGTGATGTTTAATTA~TTTTTTTCAAATTTACCCTTGATATATACCTA

*Pain1-8a* CGTCCAAAATAATTGATGTTTCACATAATCAACGTGATGTTTAAA~~TTTTTTTTCAAATTTACCCTTGATATATACCTA

*Pain1-8b* CGTCCAAAATAATTGATGTTTCACATAATCAACGTGATGTTTAATTTTTTTTTTTCAAATTTACCCTTGATATATACCTA

*Pain1-8d* CGTCCAAAATAATTGATGTTTCACATAATCAACGTGATGTTTAATTTTTTTTTTTCAAATTTACCCTTGATATATACCTA

*Pain1-8c* ATCCCTATAATGATTATGCCAAATCTAATATGAAAAGAAAATCATAATTACAGATATTTTAGTCACAATTAATTCATGTT

*Pain1-8a* ATCCCTATAATGATTATGCCAAATCTAATATGAAAAGAAAATCATAATTACAGATATTTTAGTCACAATTAATTCATGTT

*Pain1-8b* ATCCCTATAATGATTATGCCAAATCTAATATGAAAAGAAAATCATAATTACAGATATTTTAGTCACAATTAATTCATGTT

*Pain1-8d* ATCCCTATAATGATTATGCCAAATCTAATATGAAAAGAAAATCATAATTACAGATATTTTAGTCACAATTAATTCATGTT

*Pain1-8c* AAAATATCAATAATTTTGGATTGGAGGGAGTACTAATTAGGAAAATAATTAAGTTAAATCATTTTCACTAAACATTGTTT

*Pain1-8a* AAAATATCAATAATTTTGGATTGGAGGGAGTACTAATTAGGAAAATAATTAAGTTAAATCATTTTCACTAAACATTGTTT

*Pain1-8b* AAAATATCAATAATTTTGGATTGGAGGGAGTACTAATTAGGAAAATAATTAAGTTAAATCATTTTCACTAAACATTGTTT

*Pain1-8d* AAAATATCAATAATTTTGGATTGGAGGGAGTACTAATTAGGAAAATAATTAAGTTAAATCATTTTCACTAAACATTGTTT

*Pain1-8c* AGACTAAGGATGAAATAGGGGAGGAATCAATTATCTTATTTTTGTAAATGGATAAGTATTTTGAAATAACAAATTTTAAG

*Pain1-8a* AGACTAAGGATGAAATAGGGGAGGAATCAATTATCTTATTTTTGTAAATGGATAAGTATTTTGAAATAACAAATTTTAAG

*Pain1-8b* AGACTAAGGATGAAATAGGGGAGGAATCAATTATCTTATTTTTGTAAATGGATAAGTATTTTGAAATAACAAATTTTAAG

*Pain1-8d* AGACTAAGGATGAAATAGGGGAGGAATCAATTATCTTATTTTTGTAAATGGATAAGTATTTTGAAATAACAAATTTTAAG

*Pain1-8c* AAAACACGACAAGTCAAATAGAGTAGGATTTGATGGAGTGTATTCTAACCTTTCTAGATATTCATAAAAATTGGTTGAAT

*Pain1-8a* AAAACACGACAAGTCAAATAGAGTAGGATTTGATGGAGTGTATTCTAACCTTTCTAGATATTCATAAAAATTGGTTGAAT

*Pain1-8b* AAAACACGACAAGTCAAATAGAGTAGGATTTGATGGAGTGTATTCTAACCTTTCTAGATATTCATAAAAATTGGTTGAAA

*Pain1-8d* AAAACACGACAAGTCAAATAGAGTAGGATTTGATGGAGTGTATTCTAACCTTTCTAGATATTCATAAAAATTGGTTGAAC

*Pain1-8c* TTTTTTTAATAAACACGACAAGTTGATGAATTAGGCTTGTTGTTCCAATATAATTGGGATTAACATGAGATCGTGTGGCA

*Pain1-8a* TTTTTTTAATAAACACGACAAGTTGATGAATTAGGCTTGTTGTTCCAATATAATTGGGATTAACATGAGATCGTGTGGCA

*Pain1-8b* TTTTTTTAATAAACACGACAAGTTGATGAATTAGGCTTGTTGTTCCAATATAATTGGGATTAACATGAGATCGTGTGGCA

*Pain1-8d* AAATTTTAATAAACACGACAAGTTGATGAATTAGGCTTGTTGTTCCAATATAATTGGGATTAACATGAGATCGTGTGACA

*Pain1-8c* GGAAAGTTTTTTGGTTTTGGGTAATTTTCCAATAAAAATTAAACACATGATTGGTCAGTTTTATACAAGTTTGGAAATCA

*Pain1-8a* GGAAAGTTTTTTGGTTTTGGGTAATTTTCCAATAAAAATTAAACACATGATTGGTCAGTTTTATACAAGTTTGGAAATCA

*Pain1-8b* GGAAAGTTTTTTGGTTTTGGGTAATTTTCCAATAAAAATTAAACACATGATTGGTCAGTTTTATACAAGTTTGGAAATCA

*Pain1-8d* GCAATGTTTTTTGGTTTTGGGTAATTTTCCAATAAAAATTAAACACATGATTGGTCAGTTTTACACAAGTTTGGAAACCA

*Pain1-8c* ATCACGTTATGTGGGTCATACTTTTTTGTAGTAATGTAATAATTCCATTAGTTGGCCCCCCATCCAAATTATTTGTCCAT

*Pain1-8a* ATCACGTTATGTGGGTCATACTTTTTTGTAGTAATGTAATAATTCCATTAGTTGGCCCCCCATCCAAATTATTTGTCCAT

*Pain1-8b* ATCACGCTATGTGGGTCATACTTTTTTGTAGTAATGTAATAATTCCATTAGTTGGCCCCCCATCCAAATTATTTGTCTAC

*Pain1-8d* ATCACGTTATGTGGATCATACTTTTTTGTAGTAATGTAATAATTCCATTAGTTGGGCCCCCACCCAAATTATTTATCCAT

*Pain1-8c* CTTTCCACTTGGTCATTTTCTCTTCTTTTATTTTTTTGAAATGGAGTAGGTTATCTTGTGCCGGTTAGAAGCAATTACTA

*Pain1-8a* CTTTCCACTTGGTCATTTTCTCTTCTTTTATTTTTTTGAAATGGAGTAGGTTATCTTGTGCCGCTTAGAAGCAATTACTA

*Pain1-8b* CTTTCCACTTGGTCATTTTCTCTTCTTTTATTTTTTTGAAATGGAGTAGGTTATCTTGTGCCGCTTAGAAGCAATTACTA

*Pain1-8d* CTTTCCACTTGGTCATT~~~~~~~~TTTTTTTTTTTTGAAATGGAGTAGGTTATCTTG~GCCGCTT~~~AGCAATTACTA

*Pain1-8c* TTACCATTTCGAAGTCA~~~~TAAAAAAATCAATATATATCATATGGATAAAAATATATAACATAAATTTCATGAGTTTA

*Pain1-8a* TTACCATTTCGAAGTCA~~~~TAAAAAAATCAATATATATCATATGGATAAAAATATATAACATAAATTTCATGAGTTTA

*Pain1-8b* TTACCATTTCGAAGTCA~~~~TAAAAAAATCAATATATATCATATGGATAAAAATATATAACATAAATTTCATGAGTTTA

*Pain1-8d* TTACCATTTGGAAGTCATCTCTAAAAAAATCAATATATATCATATGGATAAAAATATATAACATAAATTTCATGAGTTTA

*Pain1-8c* TTTATTTAAATTTTAGGGGAGGAGGACATAACATAGTAACATATCACTAGTAAAATTTGTTTAAGTAGCTTGTTGAAGAT

*Pain1-8a* TTTATTTAAATTTTAGGGGAGGAGGACATAACATAGTAACATATCACTAGTAAAATTTGTTTAAGTAGCTTGTTGAAGAT

*Pain1-8b* TTTATTTAAATTTTAGGGGAGGAGGACATAACATAGTAACATATCACTAGTAAAATTTGTTTAAGTAGCTTGTTGAAGAT

*Pain1-8d* TTTATTTAAATTTTAGGGGAGGAGAACATAACATAGTAACATATCACTAGTAAAATTTGTTTACGTAGCTTGTTGAAGAT

*Pain1-8c* AATCTTAATTATCCAAAAGTAAAAAATAATAACTCATGGCGAAATTTTCAATAAAAGAACGTTATCTTTTTGCCGCAAAA

*Pain1-8a* AATCTTAATTATCCAAAAGTCAAAAATAATAACTCATGGCGAAATTTTCAATAAAAGAACGTTATCTTTTTGCCGCAAAA

*Pain1-8b* AATCTTAATTATCCAAAAGTCAAAAATAATAACTCATGGCGAAATTTTCAATAAAAAAAGGTTATCTTTTTGCCGCAAAA

*Pain1-8d* AATC~~~~TTATCCAAAAGTCAAAAATAATAACTTGTGGCGAAATTTTTAATAAAAGAACGTTATCTTTTTGCCGCAAAA

*Pain1-8c*  AGCATAGCAATTTTGGTACGGAACATATTGAGATTTCGTAGAGTATTTTACAATTCAAATTGCATAGAAAAGTCTTACCT

*Pain1-8a* AGCATAGCAATTTTGGTACGGAACATATTGAGATTTCGTACAGTATTTTACAATTCAAATTGCATAGAAAAGTCTTACCT

*Pain1-8b* AGCATAGCAATTTTGGTACGGAACATATTGAGATTTCGTACAGTATTTTACAATTTAAATTGCATAGAAAAGTCTTACCT

*Pain1-8d* AGCATAGCAATTTTGGTACGGAACATATTGAGATTTCGTAGAGTATTTTACAATTCGAATTGCATAGAAAAGTCTTACCT

*Pain1-8c* AATGCAAGTAAAATACATAATTACTTTGAAATTTCTACTAACGTGAATAAATTGGTCAACAGGTCCATTGTACCACAAGG

*Pain1-8a* AATGCAAGTAAAATACATAATTACTTTGAAATTTCTACTAACGTGAATAAATTGGTCAACAGGTCCATTGTACCACAAGG

*Pain1-8b* AATGTAAGTAAAATACATAATTACTTTGAAATTTCTACTAACTTGAATAAATTGGTCAACAGGTCCATTGTACCACAAGG

*Pain1-8d* .ATGCAAGTAAAATACATAATTACTTTGAAATTTCTACTAACGTGAATAAATTGGTTAACAGGTCCATTGTACCACAAGG

*Pain1-8c*  GATGGTATCATCTTTTTTATCAATACAATCCAGATTCAGCTATTTGGGGAAATATCACATGGGGCCATGCCGTATCCAAG

*Pain1-8a* GATGGTATCATCTTTTTTATCAATACAATCCAGATTCAGCTATTTGGGGAAATATCACATGGGGCCATGCCGTATCCAAG

*Pain1-8b*  GATGGTATCATCTTTTTTATCAATACAATCCAGATTCAGCTATTTGGGGAAATATCACATGGGGCCATGCCGTATCCAAG

*Pain1-8d*  GATGGTATCATCTTTTTTATCAATACAATCCAGATTCAGCTATTTGGGGAAATATCACATGGGGCCATGCCGTATCC~AG

*Pain1-8c* GACTTGATCCACTGGCTCTACTTGCCTTTTGCCATGGTTCCTGATCAATGGTACGATATTAACGGTGTCTGGACTGGGT

*Pain1-8a* GACTTGATCCACTGGCTCTACTTGCCTTTTGCCATGGTTCCTGATCAATGGTACGATATTAACGGTGTCTGGACTGGGT

*Pain1-8b* GACTTGATCCACTGGCTCTACTTGCCTTTTGCCATGGTTCCTGATCAATGGTACGATATTAACGGTGTCTGGACTGGGT

*Pain1-8d* GACTTGATCCACTGGCTCTACTTGCCTTTTGCCATGGTTCCTGATCAATGGTACGATATTAACGGTGTCTGGACTGGGT

***StpL***

*StpL-3h* GTTCAGAGACATCATGGCAACTTTTGCTGTCTCTGGATTGAACTCAATTTCAAGTATTTCTAGTTTTAACAACAATTTCA

*StpL-3b* GTTCAGAGACATCATGGCAACTTTTGCTGTCTCTGGATTGAACTCAATTTCAAGTATTTCTAGTTTTAACAACAATTTCA

*StpL-3f* GTTCAGAGACATCATGGCAACTTTTGCTGTCTCTGGATTGAACTCAATTTCAAGTATTTCTAGTTTTAATAACAATTTCA

*StpL-3a* GTTCAGAGACATCATGGCAACTTTTGCTGTCTCTGGATTGAACTCAATTTCAAGTATTTCTAGTTTTAATAACAATTTCA

*StpL-3d* GTTCAGAGACATCATGGCAACTTTTGCTGTCTCTGGATTGAACTCAATTTCAAGTATTTCTAGTTTTAATAACAATTTCA

*StpL-3g* GTTCAGAGACATCATGGCAACTTTTGCTGTCTCTGGATTGAACTCAATTTCAAGTATTTCTAGTTTTAATAACAATTTCA

*StpL-3e* GTTCAGAGACATCATGGCAACTTTTGCTGTCTCTGGATTGAACTCAATTTCAAGTATTTCTAGTTTTAATAACAATTTCA

*StpL-3c* GTTCAGAGACATCATGGCAACTTTTGCTGTCTCTGGATTGAACTCAATTTCAAGTATTTCTAGTTTTAATAACAATTTCA

*StpL-3h* GAAGCAAAAACTCAAACATTTTGTTGAGTAGAAGGAGGATTTTATTGTTCAGTTTTAGAAGAAGAAGAAGAAGTTTCTCT

*StpL-3b* GAAGCAAAAACTCAAACATTTTGTTGAGTAGAAGGAGGATTTTATTGTTCAGTTTTAGAAGAAGAAGAAGAAGTTTCTCT

*StpL-3f* GAAGCAAAAACTCAAACATTTTGTTGAGTAGAAGGAGGATTTTATTGTTCAGTTTTAGAAGAAGAAGAAG~~~TTTCTCT

*StpL-3a* GAAGCAAAAACTCAAACATTTTGTTGAGTAGAAGGAGGATTTTATTGTTCAGTTTTAGAAGAAGAAGAAG~~~TTTCTCT

*StpL-3d* GAAGCAAAAACTCAAACATTTTGTTGAGTAGAAGGAGGATTTTATTGTTCAGTTTTAGAAGAAGAAGAAGAAGTTTCTCT

*StpL-3g* GAAGCAAAAACTCAAACATTTTGTTGAGTAGAAGGAGGATTTTATTGTTCAGTTTTAGAAGAAGAAGAAGAAGTTTCTCT

*StpL-3e* GAAGCAAAAACTCAAACATTTTGTTGAGTAGAAGGAGGATTTTATTGTTCAGTTTTAAAAGAAGAAGAAGAAGTTTCTCT

*StpL-3c* GAAGCAAAAACTCAAACATTTTGTTGAGTAGAAGGAGGATTTTATTGTTCAGTTTTAGAAGAAGAAGAAGAAGTTTCTCT

*StpL-3h* GTTAGCAACGTTGCTAGCGATCAAAAGCAGAAGACTAAGGATTCTTCCTCTGATGAAGGTACTTGAAATAATTTTACGTT

*StpL-3b* GTTAGCAACGTTGCTAGCGATCAAAAGCAGAAGACTAAGGATTCTTCCTCTGATGAAGGTACTTGAAATAATTTTACGTT

*StpL-3f* GTTTGCAATGTTGCTAGTGATCAAAAGCAGAAGACTAAGGATTCTTCCTCTGATGAAGGTACTTGAAATAATTTTCCGTT

*StpL-3a* GTTTGCAATGTTGCTAGTGATCAAAAGCAGAAGACTAAGGATTCTTCCTCTGATGAAGGTACTTGAAATAATTTTCCGTT

*StpL-3d* GTTAGCAATGTTGCTAGTGATCAAAAGCAGAAGACTAAGGATTCTTCCTCTGATGAAGGTACTTGAAATAATTTTCCGTT

*StpL-3g* GTTAGCAATGTTGCTAGTGATCAAAAGCAGAAGACTAAGGATTCTTCCTCTGATGAAGGTACTTGAAATAATTTTCCGTT

*StpL-3e* GTTAGCAGTGTTGCTAGTGATCAAAAGCAGAAGACAAAGGATTCTTCCTCTGATGAAGGTACTTGAAATAATTTTCCGTT

*StpL-3c* GTTAGCAGTGTTGCTAGTGATCAAAAGCAGAAGACAAAGGATTCTTCCTCTGATGAAGGTACTTGAAATAATTTTCCGTT

*StpL-3h* CAATTTATTTGTCTGGTTTTAACTTGACACGGAGTTTAAAAAAGTAAAGACCTGTTGTGGTATGAAATCGAAGATATGTT

*StpL-3b* CAATTTATTTGTCTGGTTTTAACTTGACACGGAGTTTAAAAAAGTAAAGACCTGTTGTGGTATGAAATCGAAGATATGTT

*StpL-3f* CAATTTATTTGTCTGGTTTTAACTTGACACGGAGTTTAAAAAAGTAAAGACCTGTTGTGGTATGAAATCGAAGATATGTT

*StpL-3a* CAATTTATTTGTCTGGTTTTAACTTGACACGGAGTTTAAAAAAGTAAAGACCTGTTGTGGTATGAAATCGAAGATATGTT

*StpL-3d* CAATTTATTTGTCTGGTTTTAACTTGACATGGAGTTTAAAAAAGTAAAGACCTATTGTGGTATGAAATCGAAGATATGTT

*StpL-3g* CAATTTATTTGTCTGGTTTTAACTTGACACGGAGTTTAAAAAAGTAAAGACCTGTTGTGGTATGAAATCGAAGATATGTT

*StpL-3e* CAATTTATTTGTCTGGTTTTGACTTGATACGGAGTTTAAGAAAGTAAAGACCTGTTGTGGTATGAAATCGAAGATATGTT

*StpL-3c* TAATTTATTTGTCTGGTTTTGACTTGATACGGAGTTTAAGAAAGTAAAGACCTGTTGTGGTATGAAATCGAAGATATGTT

*StpL-3h* GAATGTACTAAAATGTTCTTTAATCTTGTGGTGTTAAACGTACTAGGTGAAAAGTTAAAAAATTAAAGAGTTGTCATAAA

*StpL-3b* GAATGTACTAAAATGTTCTTTAATCTTGTGGTGTTAAACGTACTAGGTGAAAAGTTAAAAAATTAAAGAGTTGTCATAAA

*StpL-3f* GAATGTACTAAAATGTTCTTTAATCTTGTGGTGTTAAACGTACCAGGTGAAAAGTTAAAAAATTAAAGAGTTGTCATAAA

*StpL-3a* GAATGTACTAAAATGTTCTTTAATCTTGTGGTGTTAAACGTACCAGGTGAAAAGTTAAAAAATTAAAGAGTTGTCATAAA

*StpL-3d* GAATGTACTAAAATGTTCTTTAATCTTGTGGTGTTAAACGTACTAGGTGAAAAGTTAAAAAATTAAAGAGTTGTCATAAA

*StpL-3g* GAATGTACTAAAATGTTCTTTAATCTTGTGGTGTTAAACGTACTAGGTGAAAAGTTAAAAAATTAAAGAGTTGTCATAAA

*StpL-3e* GAATGTACTAAAATGTTCTTTAATCTTATGGTGTTAAACGTATTAGGTGAAAAGTTAAAAAATTAAAGAGTTGTCATAAA

*StpL-3c* GAATGTACTAAAATGTTCTTTAATCTTGTGGTGTTAA~CGTATTAGGTGAAAAGTTAAAAAATTAAAGAGTTGTCATAAA

*StpL-3h* AGGGGTGAGGCATTATTTTTGAAATAGACTAGAAATGAAAGTAAGATAAACAAATTGAATGGAGAGAGTGTTAAAGTTTT

*StpL-3b* AGGGGTGAGGCATTATTTTTGAAATAGACTAGAAATGAAAGTAAGATAAACAAATTGAATGGAGAGAGTGTTAAAGTTTT

*StpL-3f* AGGGGTGAGGCATTATTTTTGAAATAGACTAGAAATGAAAGTAAGATAAACAAATTGAATGGAGAGAGTGTTAAAGTTTT

*StpL-3a* AGGGGTGAGGCATTATTTTTGAAATAGACTAGAAATGAAAGTAAGATAAACAAATTGAATGGAGAGAGTGTTAAAGTTTT

*StpL-3d* AGGGGCGAGGCATTATTTTTGAAATAGACTAGAAATGAAAGTAAGACAAACAAATTGAATGGAGAGAGTGTTAAAGTTTT

*StpL-3g* AGGGGTGAGGCATTATTTTTGAAATAGACTAGAAATGAAAGTAAGATAAACAAATTGAATGGAGAG~~TGTTAAAGTTTT

*StpL-3e* AGGGGTGAGGCATTATTTTTGAAATAGACTAGAAATGAAAGTAAGATAAACAAATTGAATGCAGAGAGTGTAAAAGTTTT

*StpL-3c* AGGGGCGAGGCATTATTTTTGAAATAGACTAGAAATGAAAGTAAGATAAACAAATTGAATGCAGAGAATGTAAAAGTTTT

*StpL-3h* GAACTTTTGGGTTCGTGTGTGTTAGAGTCTCACATTGGTTAAGGAAATGAGTTGATTCCTATACGGTATTAGCAATGCTC

*StpL-3b* GAACTTTTGGGTTCGTGTGTGTTAGAGTCTCACATTGGTTAAGGAAATGAGTTGATTCCTATACGGTATTAGCAATGCTC

*StpL-3f* GAACTTTTGGGTTCGTGTGTGTTAGAGTCTCACATTGGTTAAGGAAATGAGTTGATTCCTATACGGTATTAGCAATGCTC

*StpL-3a* GAACTTTTGGGTTCGTGTGTGTTAGAGTCTCACATTGGTTAAGGAAATGAGTTGATTCCTATACGGTATTAGCAATGCTC

*StpL-3d* GAACTTTTGGGTTCGTGTGTGTTAGAGTCTCACATTGGTTAAGGAAATGAGTTGATTCCTATACGGTATTAGCAATGCTC

*StpL-3g* GAACTTTTGGGTTCGTGTGTGTTAGAGTCTCACATTGGTTAAGGAAATGAGTTGATTCCTATACGGTATTAGCAATGCTC

*StpL-3e* GTACATTTGGGTTCGTGTGTGTTAGAATCTCACATTGGTTAAGGAACTCAATTGATTCCTATACGGTATTAGCAATGCTC

*StpL-3c* GTACATTTGGGTTCGTGTGTGTTAGAATCTCACATTGGTTAAGGAACTCAATTGATTCCTATACGGTATTAGCAATGCTC

*StpL-3h* ACCTCGTTAACTAGTTTTTGAGATTGACTTAGGTCCTAGGTCTACTAATTTGTTTAACATGGTATTAGAAAGACAACGAC

*StpL-3b* ACCTCGTTAACTAGTTTTTGAGATTGACTTAGGTCCTAGGTCTACTAATTTGTTTAACATGGTATTAGAAAGACAACGAC

*StpL-3f* ACCTCGTTAACTAGTTTTTGAGATTGACTTAGGTCCTAGGTCTACTAATTTGTTTAACATGGTATTAGAAAGACAACGAC

*StpL-3a* ACCTCGTTAACTAGTTTTTGAGATTGACTTAGGTCCTAGGTCTACTAATTTGTTTAACATGGTATTAGAAAGACAACGAC

*StpL-3d* ACCTTGTTAACTAGTTTTTGAGATTGACTTAGGTCCTAGGTCTACTAATTTGTTTAACATGGTATTAGAAAGACAACGAC

*StpL-3g* ACCTCGTTAACCAGTTTTTGAGATTGACTTAGGTCCTAGGTCTACTAATTTGTTTAACATGGTATTAGAAAGACAACGAC

*StpL-3e* ACCTCGTTAACTAGGTTTTGAGATTGACTTAGGTCCTAGGTCTACTAATTTGTTTAACATGGTATCAAAAAGATAACGAC

*StpL-3c* ACCTCGTTAACTAGGTTTTGAGATTGACTTAGGTCCTAGGTCTACTAATTTGTTTAGCATGGTATCAGAAAGATAACGAC

*StpL-3h* AACATCCCCAGTGTAATCCCAC~AGTGGGATCTGAGGAGGGATGTTGTGTACGTAGACCTTACTTCTATCCTGTGAAGGT

*StpL-3b* AACATCCCCAGTGTAATCCCAC~AGTGGGATCTGAGGAGGGATGTTGTGTACGTAGACCTTACTTCTATCCTGTGAAGGT

*StpL-3f* AACATCCCCAGTGTAATCCCAC~AGTGGGATCTGAGGAGGGATGTTGTGTACGTAGACCTTACTTCTATCCTGTGAAGGT

*StpL-3a* AACATCCCCAGTGTAATCCCAC~AGTGGGATCTGAGGAGGGATGTTGTGTACGTAGACCTTACTTCTATCCTGTGAAGGT

*StpL-3d* AACATACCCAGTGTAATCCCAC~AGTGGGATCTGAGGAGGGATGTTGTGTACGTAGACCTTACTTCTATCCTGTGAAGGT

*StpL-3g* AACATACCCAGTGTAATCCCAC~AGTGGAATCTGAGGAAGGATGTTGTGTACGTAGACCTTACTTCTATCCTGTGAAGGT

*StpL-3e* AACATACCCAGTGTAATCCCACTAGTGGAGTCTGAGGAGGGATGTTGTTTACGTAGACCTTACTTCTATCTTGTGAAGGT

*StpL-3c* AACATACCCAGTGTAATCCCACTAGTGGAGTCTGAGGAGGGATGTTGTTTACGTAGACCTTACTTCTATCCTGTGAAGGT

*StpL-3h* AGATAAAGCGTTTCTGATAGACCCTCGGCTCAAGACACATTTCTGTTTTT~GGATAATTAGTATTGAGACCTTACATTAT

*StpL-3b* AGATAAAGCGTTTCTGATAGACCCTCGGCTCAAGACACATTTCTGTTTTT~GGATAATTAGTATTGAGACCTTACATTAT

*StpL-3f* AGATAAAGCGTTTCTGATAGACCCTCGGCTCAAGACACATTTCTGTTTTT~GGATAATTAGTATTGAGACCTTACATTAT

*StpL-3a* AGATAAAGCGTTTCTGATAGACCCTCGGCTCAAGACACATTTCTGTTTTT~GGATAATTAGTATTGAGACCTTACATTAT

*StpL-3d* AGATTAGGCGTTTCTAATAGACCCTCGGCTCAAGACACATTTCTGTTTTT~GGATAATTAGTATTGAGACCTTACATTAT

*StpL-3g* AGATAAAGCATTTCTGATAGACCCTTGGCTCAAGACACATTTCTGTTTTT~GGATAATTAGTATTGAGACCTTACGTTAT

*StpL-3e* AGATAGATCGTTTCTGATAGATCCTCGGCTCAAGGCACATTTCTATTTTTAGTTTAACTAGTATTGAGACCTTATGTTAT

*StpL-3c* AGATAGATCGTTTCTGATAGATCCTCAGCTCAAGGCACATTTCTATTTTTAGTTTAACTAGTATTGAGACCTTATGTTAT

*StpL-3h* ATTATCCATGCTCTAGATGTTCAGCCTTGGACGTGCCAGGGTGTTAGAGTCGTGCATTGGTGGAGGGAATAAATTGTGTT

*StpL-3b* ATTATCCATGCTCTAGATGTTCAGCCTTGGACGTGCCAGGGTGTTAGAGTCGTGCATTGGTGGAGGGAATAAATTGTGTT

*StpL-3f* ATTATCCATGCTCTAGATGTTCAGCCTTGGACGTGCCAGGGTGTTAGAGTCGTGCATTGGTGGAGGGAATAAATTGTGTT

*StpL-3a* ATTATCCATGCTCTAGATGTTCAGCCTTGGACGTGCCAGGGTGTTAGAGTCGTGCATTGGTGGAGGGAATAAATTGTGTT

*StpL-3d* ATTATCCATGCTCTAGATGTTCAGCCTTGGACGTGCCAGGGTGTTAGAGTCGTGCATTGGTGGAGGGAATAAATTGTGTT

*StpL-3g* ATTATCCATACTCCAGATGTTCAGCCTTGGACGTGCCAGGGTGTTAGAGTCGTGCATTGGTGGAGGGAATAAATTGTGTT

*StpL-3e* ATTGTCTACGCTCTAGATGTTCAGCCTTGGACGTGCCAGGGTGTAAGAGTCGTGCATTGGTGGAGAGAGTAGGTTGTGTT

*StpL-3c* ATTGTCTACGCTCTAGATGTTCAGCCTTGGACGTGCCAAGGTGTAAGAGTCGTGCATTGGTGGAGAGAGTAGGTTGTGTT

*StpL-3h* TGTTTAGAGAGGTGGAATTTGAAAATTTCGTATATAGTGGAGTTCTCACTCCTTAACGACTATTAACATAGCGGAAGTAG

*StpL-3b* TGTTTAGAGAGGTGGAATTTGAAAATTTCGTATATAGTGGAGTTCTCACTCCTTAACGACTATTAACATAGCGGAAGTAG

*StpL-3f* TGTTTAGAGAGGTGGAATTTGAAAATTTCGTATATAGTGGAGTTCTCACTCCTTAACGACTATTAACATAGCGGAAGTAG

*StpL-3a* TGTTTAGAGAGGTGGAATTTGAAAATTTCGTATATAGTGGAGTTCTCACTCCTTAACGACTATTAACATAGCGGAAGTAG

*StpL-3d* TGTTTAGAGAGGTGGAATTTGAAAATTTCGTATATAGTGGAGTTCTCACTCCTTAACAACTATTAACATAGCGGAAGTAG

*StpL-3g* TGTTTAGAGAGGTGGAATTTGAAAATTTCGTATATAGTGGAGTTCTCACTCCTTAACGACTATTAACATAGCGGAAGTAG

*StpL-3e* TGTTTAGATAGGTGAAATTTGAAAATTTTGCATATAATGGAGTTCTCATTCCTTAACGACTATTAACATTGCCGAAGTAG

*StpL-3c* TGTTTAGATAGGTGGAATTTGAAAATTTTGTATATAATGGAGTTCTCATTCCTTAACGACTATTAACATTGCCGAAGTAG

*StpL-3h* AGAAGCAACATACATTATTTGGACCCTTTGTTGATTGGAGATCAGCAATTCCAATTGGACTTATAAATGTTTGTTTTTGG

*StpL-3b* AGAAGCAACATACATTATTTGGACCCTTTGTTGATTGGAGATCAGCAATTCCAATTGGACTTATAAATGTTTGTTTTTGG

*StpL-3f* AGAAGCAACATACATTATTTGGACCCTTTGTTGATTGGAGATCAGCAATTCCAATTGGACTTATAAATGCTTGTTTTTGG

*StpL-3a* AGAAGCAACATACATTATTTGGACCCTTTGTTGATTGGAGATCAGCAATTCCAATTGGACTTATAAATGCTTGTTTTTGG

*StpL-3d* AGAAGCAACATACATTATTTGGACCCTTTGTTGATTGGAGATCAGCAATTCCAATTGGACTTATAAATGTTTGTTTTTGG

*StpL-3g* AGAAGCAACATACATTATTTGGACCCTTTGTTGATTGGAGATCAGCAATTCCAATTGGACTTATAAATGTTTGTTTTTGG

*StpL-3e* AGAAGCAACATACATTATTTGGACCCTTTGT~~~~~~~~~~~~~~~~~~~~~~~~~~~~~~~~~~~~~~~~~~~~~~~~~

*StpL-3c* AGAAGCAACATACATTATTTGGACCCTTTGT~~~~~~~~~~~~~~~~~~~~~~~~~~~~~~~~~~~~~~~~~~~~~~~~~

*StpL-3h* TATTTTCAGGATTTACATTAGATGTTTTTCAGCCGGACTCCACGTCTGTTTTATCAAGTATAAAGTATCACGCAGAGTTC

*StpL-3b* TATTTTCAGGATTTACATTAGATGTTTTTCAGCCGGACTCCACGTCTGTTTTATCAAGTATAAAGTATCACGCAGAGTTC

*StpL-3f* TATTTTCAGGATTTACATTAGATGTTTTTCAGCCGGACTCCACGTCTGTTTTATCAAGTATAAAGTATCACGCAGAGTTC

*StpL-3a* TATTTTCAGGATTTACATTAGATGTTTTTCAGCCGGACTCCACGTCTGTTTTATCAAGTATAAAGTATCACGCAGAGTTC

*StpL-3d* TATTTTCAGGATTTACATTAGATGTTTTTCAGCCGGACTCCACGTCTGTTTTATCAAGTATAAAGTATCACGCAGAGTTC

*StpL-3g* TATTTTCAGGATTTACATTAGATGTTTTTCAGCCAGACTCCACGTCTGTTTTATCAAGTATAAAGTATCACGCAGAGTTC

*StpL-3e* ~ATTTTCAGGATTTACATTAGATGTTTTTCAGCCGGACTCCACGTCTGTTTTATCAAGTATAAAGTATCACGCTGAGTTC

*StpL-3c* ~ATTTTCAGGATTTACATTAGATGTTTTTCAGCCGGACTCCACGTCTGTTTTATCAAGTATAAAGTATCACGCTGAGTTC

*StpL-3h* ACGCCATCATTTTCTCCTGAGAAGTTTGAACTTCCCAAGGCATACTATGCAACTGCAGAGAGTGTTCGAGATACGCTCAT

*StpL-3b* ACGCCATCATTTTCTCCTGAGAAGTTTGAACTTCCCAAGGCATACTATGCAACTGCAGAGAGTGTTCGAGATACGCTCAT

*StpL-3f* ACGCCATCATTTTCTCCTGAGAAGTTTGAACTTCCCAAGGCATACTATGCAACTGCAGAGAGTGTTCGAGATACGCTCAT

*StpL-3a* ACGCCATCATTTTCTCCTGAGAAGTTTGAACTTCCCAAGGCATACTATGCAACTGCAGAGAGTGTTCGAGATACGCTCAT

*StpL-3d* ACGCCATCATTTTCTCCTGAGAAGTTTGAACTTCCCAAGGCATACTATGCAACTGCAGAGAGTGTTCGAGATACGCTCAT

*StpL-3g* ACGCCATCATTTTCTCCTGAGAAGTTTGAACTTCCCAAGGCATACTATGCAACTGCAGAGAGTGTTCGAGATACGCTCAT

*StpL-3e* ACGCCATCATTTTCTCCTGAGAAGTTTGAACTTCCCAAGGCATACTATGCAACTGCAGAGAGTGTTCGAGATACGCTCAT

*StpL-3c* ACACCATCATTTTCTCCTGAGAAGTTTGAACTTCCCAAGGCATACTATGCAACTGCAGAGAGTGTTCGAGATACGCTCAT

*StpL-3h* TATAAATTGGAATGCCACATACAAATTCTATGAAAAGATGAATGTAAAGCAGGCATATTACTTGTCTATGGAATTTCTTC

*StpL-3b* TATAAATTGGAATGCCACATACAAATTCTATGAAAAGATGAATGTAAAGCAGGCATATTACTTGTCTATGGAATTTCTTC

*StpL-3f* TATAAATTGGAATGCCACATACAAATTCTATGAAAAGATGAATGTAAAGCAGGCATATTACTTGTCTATGGAATTTCTTC

*StpL-3a* TATAAATTGGAATGCCACATACAAATTCTATGAAAAGATGAATGTAAAGCAGGCATATTACTTGTCTATGGAATTTCTTC

*StpL-3d* TATAAATTGGAATGCCACATACAAATTCTATGAAAAGATGAATGTAAAGCAGGCATATTACTTGTCTATGGAATTTCTTC

*StpL-3g* TATAAATTGGAATGCCACATACGAATTCTATGAAAAGATGAATGTAAAGCAGGCATATTACTTGTCTATGGAATTTCTTC

*StpL-3e* TATAAATTGGAATGCCACATACGAATTCTATGAAAAGATGAATGTAAAGCAGGCATATTATTTGTCTATGGAATTTCTTC

*StpL-3c* TATAAATTGGAATGCCACATACGAATTCTATGAAAAGATGAATGTAAAGCAGGCATATTACTTGTCTATGGAATTTCTTC

*StpL-3h* AGGTTAGATTAGCTAGGCTTCCCCTTTATTTCGAGTATAACTACAGACGTCACTCGTTGGAGTGAGTTGCTTTAATATGC

*StpL-3b* AGGTTAGATTAGCTAGGCTTCCCCTTTATTACGAGTATAACTACAGACGTCACTCGTTGGAGTGAGTTGCTTTAATATGC

*StpL-3f* AGGTTAGATTAGCTAGGCTTCCCCTTTATTTCGAGTATAACT~CAGACGTCACTCGTTGGAGTGAGTTGCTTTAATATGC

*StpL-3a* AGGTTAGATTAGCTAGGCTTCCCCTTTATTTCGAGTATAACT~CAGACGTCACTCGTTGGAGTGAGTTGCTTTAATATGC

*StpL-3d* AGGTTAGATTAGCTAGGCTTCCCCTTTATTTCGAGTATAACTACAGACGTCACTCGTTGGAGTGAGTTGCTTTAATATGC

*StpL-3g* AGGTTAGATTAGCTAGGCTTCCCCTTTATTTCGAGTATAACTACAGACGCCACTCGTTGGAGTGAGTTGCTTTAATATGC

*StpL-3e* AGGTTAGATTAGCTAGGCTTCCCCTTTATTTCGAGTATAACTACAGACGTCACTCGTTGGAGTGAGTTGCTTTAATATGC

*StpL-3c* AGGTTAGATTAGCTAGGCTTCCCCTTTATTTTGAGTATAACTACAGACGTCACTCGTTGGAGTGAGTTGCTTTAATATGC

*StpL-3h* TTTTGGTGCTTGCAGGGAAGAGCTTTACTCAATGCTATTGGTAACTTGGGGCTAACCGGACCTTATGCAGATGCTTTAAC

*StpL-3b* TTTTGGTGCTTGCAGGGAAGAGCTTTACTCAATGCTATTGGTAACTTGGGGCTAACCGGACCTTATGCAGATGCTTTAAC

*StpL-3f* TTTTGGTGCTTGCAGGGAAGAGCTTTACTCAATGCTATTGGTAACTTGGGGCTAACCGGACCTTATGCAGATGCTTTAAC

*StpL-3a* TTTTGGTGCTTGCAGGGAAGAGCTTTACTCAATGCTATTGGTAACTTGGGGCTAACCGGACCTTATGCAGATGCTTTAAC

*StpL-3d* TTTTGGTGCTTGCAGGGAAGAGCTTTACTCAATGCTATTGGTAACTTGGGGCTAACCGGACCTTATGCAGATGCTTTAAC

*StpL-3g* TTTTGGTGCTTGCAGGGAAGAGCTTTACTCAATGCTATTGGTAACTTGGGGCTAACCGGACCTTATGCAGATGCTTTAAC

*StpL-3e* TTTTGGTGCTTTCAGGGAAGAGCTTTACTCAATGCTATTGGTAACTTGGGGCTAACCGGACCTTATGCAGATGCTTTAAC

*StpL-3c* TTTTGATGCTTGCAGGGAAGAGCTTTACTCAATGCTATTGGTAACTTGGGGCTAACCGGACCTTATGCAGATGCTTTAAC

*StpL-3h* TAAGCTCGGATACAGTTTAGAGGATGTAGCCAGGCAGGTCAGGTTTCTGTAGGAACTTACTTATTTCTGTTGACGGTTAG

*StpL-3b* TAAGCTCGGATACAGTTTAGAGGATGTAGCCAGGCAGGTCAGGTTTCTGTAGGAACTTACTTATTTCTGTTGACGGTTAG

*StpL-3f* TAAGCTCGGATACAGTTTAGAGGATGTAGCCAGGCAGGTCAGGTTTCTGTAGGAACTTACTTATTTCTGTTGATGATTAG

*StpL-3a* TAAGCTCGGATACAGTTTAGAGGATGTAGCCAGGCAGGTCAGGTTTCTGTAGGAACTTACTTATTTCTGTTGATGATTAG

*StpL-3d* TAAGCTCGGATACAGTTTAGAGGATGTAGCCAGGCAGGTCAGGTTTCTGTAGGAACTTACTTATTTCTGTTGATGGTTAG

*StpL-3g* TAAGCTCGGATACAGTTTAGAGGATGTAGCCAGGCAGGTCGGGTTTCTGTAGGAACTTACTTATTTCTGTTGACGGTTAG

*StpL-3e* TAAGCTCGGATACAGTTTAGAGGATGTAGCCAGGCAGGTCGGGTTTCTGTAGGAACTTACTTATTTCTGTTGACGGTTAG

*StpL-3c* TAAGCTCGGATACAGTTTAGAGGATGTAGCCAGGCAGGTCGGGTTTCTGTAGGAACTTACTAATTTCTGTTGACGGTTAG

*StpL-3h* CCACTCGAAGTAGTTTTTGTTCAATTGAGTCTTTGTCTTGCCTTGTTTGTCTATTACTAACACTGAAACTCTGCTCTAAA

*StpL-3b* CCACTCGAAGTAGTTTTTGTTCAATTGAGTCTTTGTCTTGCCTTGTTTGTCTATTACTAACACTGAAACTCTGCTCTAAA

*StpL-3f* TCACTCGAAGTAGTTTTTGTTCAATTGAGTCTTTGTCTTGCCTTGTTTGTCTATTACTAACACTGAAACTCTGCTCTAAA

*StpL-3a* TCACTCGAAGTAGTTTTTGTTCAATTGAGTCTTTGTCTTGCCTTGTTTGTCTATTACTAACACTGAAACTCTGCTCTAAA

*StpL-3d* CCACTCGAAGTAGTTTTTGTTCAATTGAGTCTTTGTCTTGCCTTGTTTGACTATTACTAACACTGAAACTCTGCTCTAAA

*StpL-3g* CCACTTGAAGTAGTTTTTGTTCAATTGAGTCTTTGTCTTGCCTTGTTTGTCTATTACTAACACTGAAACTCTGCTCTAAA

*StpL-3e* CCACTCGAAGTAGTTTTTGTTCAATTGAGTCTTTGTCTTGCCTTGTTTGACTATTACTAACACTGAAACTCTGCTCTAAA

*StpL-3c* CCACTCGAAGTAGTTTTTGTTCAATTGAGTCTTTG~CTTGCCTTGTTTGACTATTACTAACACT~TAACTCTGCTCCAAA

*StpL-3h* ATGAGAAATGTTGAAGGCGACTAGCTACCGTTTCTAGATTGCTCAAGTTCTTGATTGATATGCTTTTCCAAAATGATTTA

*StpL-3b* ATGAGAAATGTTGAAGGCGACTAGCTACCGTTTCTAGATTGCTCAAGTTCTTGATTGATATGCTTTTCCAAAATGATTTA

*StpL-3f* ATGAGAAATGTTGAAGGCGACTAGCTACCGTTTCTAGATTGCACAAGTTCTTGATTGATATGCTTTTCCAAAATGATTTA

*StpL-3a* ATGAGAAATGTTGAAGGCGACTAGCTACCGTTTCTAGATTGCACAAGTTCTTGATTGATATGCTTTTCCAAAATGATTTA

*StpL-3d* ATGAGAAATGTTGAAGGCGACTAGCTACCGTTTCTAGATTGCACAAGTTCTTGATTGATATGCTTTTCCAAAATGATTTA

*StpL-3g* ATGAGAAATGTTGAAGGCGACTAGCTCCCGTTTCTAGATTGCACAAGTTCTTGATTGATATGCTTTTCCAAAATGATTTA

*StpL-3e* ATGAGAAATGTTGAAGGCGACTAGCTACCGTTTCTAGATTGCACAAGTTCTTGATTGATATGCTTTTCCAAAATGATTTA

*StpL-3c* ATGAGAAATGTTGAAGGCGACTAGCTCCCGTTTCTAGATTGCACAAGTTCTTGATTGATATGCTTTTCCAAAATGATTTA

*StpL-3h* ATAACACGTCAACGTAGTCAAACTATCCCACTCTCATCACCACTGTTCTAACATTGTGATATATGTCAATTGATTTCACT

*StpL-3b* ATAACACGTCAACGTAGTCAAACTATCCCACTCTCATCACCACTGTTCTAACATTGTGATATATGTCAATTGATTTCACT

*StpL-3f* ATAACACGTCAACGTAGTCAAACTATCCCACTCTCATCACCACCGTTCTAACATTGTGATATATGTCAATTGATTTCACT

*StpL-3a* ATAACACGTCAACGTAGTCAAACTATCCCACTCTCATCACCACCGTTCTAACATTGTGATATATGTCAATTGATTTCACT

*StpL-3d* ATAACACGTCAACGTAGTCAAACTATCCCACTCTCATCACCACTGTTCTAACATTGTGATATATGTCAATTGATTTCACT

*StpL-3g* ATAACATGTCAACGTAGTCAAACTATCCCACTTTCATCACCACTGTTCTAACATTGTGATATATGTCAATTGATTTCACT

*StpL-3e* ATAACACGTCAACGTAGTCAAACTATCCCACTCTCATCACCACTGTTCTAACATTGTGATATATGTCAATTGATTTCACT

*StpL-3c* ATAACACGTCAACGTAGTCAAACTATCCCACTCTCATCACCACTGTTCTAACATTGTGATATATGTCAATTGATTTCACT

*StpL-3h* CGGGATAGTAGTCTACTGTAATTAGCTTAAGCTGTTTACTGTAATGACTGACAGGAACCGGATGCAGCTTTAGGTAATGG

*StpL-3b* CGGGATAGTAGTCTACTGTAATTAGCTTAAGCTGTTTACTGTAATGACTGACAGGAACCGGATGCAGCTTTAGGTAATGG

*StpL-3f* CGGGATAGTAGTCTACTGTAATTAGCTTAAGCTGTTTACTGTAATGACTGACAGGAACCGGATGCAGCTTTAGGTAATGG

*StpL-3a* CGGGATAGTAGTCTACTGTAATTAGCTTAAGCTGTTTACTGTAATGACTGACAGGAACCGGATGCAGCTTTAGGTAATGG

*StpL-3d* CGGGATAGTAGTCTACTGTAATTAGCTTAAGCTGTTTACTGTAATGACTGACAGGAACCGGATGCAGCTTTAGGTAATGG

*StpL-3g* CGGGATAGTAGTCTACTGTAATTAGCTTAAGCTGTTTACTGTAATGACTGACAGGAACCGGATGCAGCTTTAGGTAATGG

*StpL-3e* CGGGATAGTAGTCTACTGTAATTAGCTTAAGCTGTTTACTGTAATGACTGACAGGAACCGGATGCAGCTTTAGGTAATGG

*StpL-3c* CGGGATAGTAGTCTACTGTAATTAGCTTAAGCTGTTTACTGTAATGACTGACAGGAACCGGATGCAGCTTTAGGTAATGG

*StpL-3h* AGGTTTAGGAAGACTTGCTTCTTGCTTTCTGGACT

*StpL-3b* AGGTTTAGGAAGACTTGCTTCTTGCTTTCTGGACT

*StpL-3f* AGGTTTAGGAAGACTTGCTTCTTGCTTTCTGGACT

*StpL-3a* AGGTTTAGGAAGACTTGCTTCTTGCTTTCTGGACT

*StpL-3d* AGGTTTAGGAAGACTTGCTTCTTGCTTTCTGGACT

*StpL-3g* AGGTTTAGGAAGACTTGCTTCTTGCTTTCTGGACT

*StpL-3e* AGGTTTAGGAAGACTTGCTTCTTGCTTTCTGGACT

*StpL-3c* AGGTTTAGGAAGACTTGCTTCTTGCTTTCTGGACT

***Pain-1 promoter***

Pain1-Db1 TGGTCGACCTGCAGGCGGCCGCGAATTCACTAGTGATTGACCATACGTGGCTGACAAAATTCACCATTTGTTATCTTTTT

Pain1-Sb2 TGGTCGACCTGCAGGCGGCCGCGAATTCACTAGTGATTGACCATACGTGGCTGACAAAATTCACCATTTGTTATCTTTTT

Pain1-Db2 TGGTCGACCTGCAGGCGGCCGCGAATTCACTAGTGATTGACCATACGTGGCTGACAAAATTCACCATTTGTTATCTTTTT

Pain1-Sb3 TGGTCGACCTGCAGGCGGCCGCGAATTCACTAGTGATTGACCATACGTGGCTGACAAAATTCACCATTTGTTATCTTTTT

Pain1-Da TGGTCGACCTGCAGGCGGCCGCGAATTCACTAGTGATTGACCATACGTGGCTGACAAAATTCACCATTTGTTATCTTTTT

Pain1-Sa TGGTCGACCTGCAGGCGGCCGCGAATTCACTAGTGATTGACCATACGTGGCTGACAAAATTCACCATTTGTTATCTTTTT

Pain1-Dc TGGTCGACCTGCAGGCGGCCGCGAATTCACTAGTGATTGACCATACGTGGCTGACAAAATTCACCATTTGTTATCTTTTT

Pain1-Sb1 TGGTCGACCTGCAGGCGGCCGCGAATTCACTAGTGATTGACCATACGTGGCTGACAAAATTCACCATTTGTTATTTTTTT

Pain1-Db1 TGTTAGCTCCAAAAAAA-GAATTCCATAAAATCTAACACTATTTTCTTTACTTTCTCCAACAAAATTTATTATTTTATCT

Pain1-Sb2 TGTTAGCTCCAAAAAAA-GAATTCCATAAAATCTAACACTATTTTCTTTACTTTCTCCAACAAAATTTATTATTTTATCT

Pain1-Db2 TGTTAGCTCCAAAAAAAAGAATTCCATAAAATCTAACACTATTTTCTTTACTTTCTCCAACAAAATTTATTATTTTCTCT

Pain1-Sb3 TGTTAGCTCCAAAAAAA-GAATTCCATAAAATCTAACACTATTTTCTTTACTTTCTCCAACAAAATTTATTATTTTCTCT

Pain1-Da TGTTAGCTCCAAAAAAA-GAATTCCATAAAATCTAACACTATTTTCTTTACTTTCTCCAACAAAATTTATTATTTTCTCT

Pain1-Sa TGTTAGCTCCAAAAAAA-GAATTCCATAAAATCTAACACTATTTTCTTTACTTTCTCCAACAAAATTTATTATTTTCTCT

Pain1-Dc TGTTAGCTCCAAAAAAA-GAATTCCATAAAATCTAACACTATTTTCTTTACTTTCTCCAACAAAATTTATTATTTTCTCT

Pain1-Sb1 TGTTAGCTCCAAAAAAA-GAATTCCATAAAATCTAACACTATTTTCTTTACTTTCTCCAACAAAATTTATTATTTTCTCT

Pain1-Db1 TTCAAAATATCATTCCACTGACTCAATACTCATTTATGTTTTAAGCAAGAGAAAATAATAAATTTTGTTAGAGAAAGTA-

Pain1-Sb2 TTCAAAATATCATTCCACTGACTCAATACTCATTTATGTTTTAAGCAAGAGAAAATAATAAATTTTGTTAGAGAAAGTA-

Pain1-Db2 TTCAAAATATCATTCCACTAACTCAATACTCATTTATGTTTTAAGCAAGAGAAAATAATAAATTTTGTTAGAGAAAGTA-

Pain1-Sb3 TTCAAAATATCATTCCACTGACTCAATACTCATTTATGTTTTAAGCAAGAGAAAATAATAAATTTTGTTAGAGAAAGTAC

Pain1-Da TTCAAAATATCATTCCACTGACTCAATACTCATTTATGTTTTAAGCAAGGGAAAATAATAAATTTTGTTAGAGAAAGTAC

Pain1-Sa TTCAAAATATCATTCCACTGACTCAATACTCATTTATGTTTTAAGCAAGAGAAAATAATAAATTTTGTTAGAGAAAGTA-

Pain1-Dc TTCAAAATATCATTCCACTAACTCAATACTCATTTATGTTTTAAGCAAGAGAAAATAATAAATTTTGTTAGAGAAAGTA-

Pain1-Sb1 TTCAAAATATCATTCCACTGACTCAATA---ATTTCTGTTTTAAGCAAGAGAAAATAATAAATTTTGTTAGAGAAAGTA-

Pain1-Db1 ---GAAACAATGACATTAGATTTCATGGAGCTTTCATTAGAGCTAACAAAGCGAAGACAACAATGGTGATTTTGTTGGCC

Pain1-Sb2 ---GAAACAATGACATTAGATTTCATGGAGCTTTCATTAGAGCTAACAAAGCGAAGACAACAATGGTGATTTTGTTGGCC

Pain1-Db2 ---GAAACAATGACATTAGATTTCATGGAGCTTTCATTAGAGCTAACAAAGCGAAGACAACAATGGTGATTTTGTTGGCC

Pain1-Sb3 GTAGAAACAATGACATTAGATTTCATGGAGCTTTCATTAGAGCTAACAAAGCGAAGACAACAATGGTGATTTTGTTGGCC

Pain1-Da GTAGAAACAATGACATTAGATTTCATGGAGCTTTCATTAGAGCTAACAAAGCGAAGACAACAATGGTGATTTTGTTGGCC

Pain1-Sa ---GAAACAATGACATTAGATTTCATGGAGCTTTCATTAGAGCTAACAAAGCGAAGACAACAATGGTGATTTTGTTGGCC

Pain1-Dc ---GAAACAATGACATTAGATTTCATGGAGCTTTCATTAGAGCTAACAAAGCGAAGACAACAATGGTGATTTTGTTGGCC

Pain1-Sb1 ---GAAACAATGACATTAGATTTCATGGAGCTTTCATTAGAGCTAACAAAGCGAAGACAACAATGGTGATTTTGTTGGCC

Pain1-Db1 TCGTACGGTCCACGGTTAATTTAATATATATTTTTGATAAATCTATTAGGTTTATGACAAAGAACCAAAATGAACCTATT

Pain1-Sb2 TCGTACGGTCCACGGTTAATTTAATATATATTTTTGATAAATCTATTAGGTTTATGACAAAGAACCAAAATGAACCTATT

Pain1-Db2 TCGTACGGTCCACGGTTAATTTAATATATATTTTTGATAAATCTATTAGGTTTATGACACAGAACCAAAATGAACCTATT

Pain1-Sb3 TCGTACGGTCCACGGTTAATTTAATATATATTTTTGATAAATCTATTAGGTTTATGACAAAGAACCAAAATGAACCTATT

Pain1-Da TCGTACGGTCCACGGTTAATTTAATATATATTTTTGATAAATCTATTAGGTTTATGACAAAGAACCAAAATGAACCTATT

Pain1-Sa TCGTACGGTCCACGGTTAATTTAATATATATTTTTGATAAATCTATTAGGTTTATGACACAGAACCAAAATGAACCTATT

Pain1-Dc TCGTACGGTCCACGGTTAATTTAATATATATTTTTGATAAATCTATTAGGTTTATGACACAGAACCAAAATGAACCTATT

Pain1-Sb1 TCGTACGGTTCACAGTTAATTTAATATATATTTTTGATAAATCTATTAGGTTTATGAC--AGAACCAAAATGAACCTATT

Pain1-Db1 TTGTTATACATTAATAATCATTTCAATCAAGTGAAATATATTAAGGACCAAAATAAACACATTTTATATACCTTAGACCA

Pain1-Sb2 TTGTTATACATTAATAATCATTTCAATCAAGTGAAATATATTAAGGACCAAAATAAACACATTTTATATACCTTAGACCA

Pain1-Db2 TTGTTATACATTAATAATCATTTCAATCAAGTGAAATATATTAAGGACCAAAATAAACACATTTTATATACCTTAGACCA

Pain1-Sb3 TTGTTATACATTAATAATCATTTCAATCAAGTGAAATATATTAAGGACCAAAATAAACACATTTTATATACCTTAGACCA

Pain1-Da TTGTTATACATTAATAATCATTTCAATCAAGTGAAATATATTAAGGACCAAAATTAACACATTTTATATACCTTAGACCA

Pain1-Sa TTGTTATACATTAATAATCATTTCAATCAAGTGAAATATATTAAGGACCAAAATAAACACATTTTATATACCTTAGACCA

Pain1-Dc TTGTTATACATTAATAATCATTTCAATCAAGTGAAATATATTAAGGACCAAAATAAACACATTTTATATACCTTAGACCA

Pain1-Sb1 TTGTTATACATCAATAATCATTTCAATCAAGTGAAATATATTAAGGACCAAAATAAACATATTTTATATACCTTAGACCA

Pain1-Db1 TTTCAATAAAGTGAAATATATTAACGGGTCGTTTGGTGTGAAGGATAATACCAAATAATCCTGAGATTAAATTATAGTAC

Pain1-Sb2 TTTCAATAAAGTGAAATATATTAACGGGTCGTTTGGTGTGAAGGATAATACCAAATAATCCTGAGATTAAATTATAGTAC

Pain1-Db2 TTTCAATAAAGTGAAATATATTAAGGGGTCGTTTGGTGAGAAGGATAATACCAAATAATCTGAGATTAAATTATAGTACT

Pain1-Sb3 TTTCAATAAAGTGAAATATATTAAGGGGTCGTTTGGTGTGAAGGATAATACCAAATAATCCTGAGATTAAATTATAGTAC

Pain1-Da TTTCAATAAAGTGAAATATATTAAGGGGTCGTTTGGTGTGAAGGATAATACCAAATAATCCTGAGATTAAATTATAGTAC

Pain1-Sa TTTCAATAAAGTGAAATATATTAAGGGGTCGTTTGGTGAGAAGGATAATACCAAATAATCCTGAGATTAAATTATAGTAC

Pain1-Dc TTTCAATAAAGTGAAATATATTAAGGGGTCGTTTGGTGAGAAGGATAATACCAAATAATCTTGAGATTAAATTATAGTAC

Pain1-Sb1 TTTCAATAAAGTGAAATATATTAAGGGGTCGTTTGGTGTGAAGGATAATACCAAATAATCCTGAGATTAAATTATAGTAC

Pain1-Db1 CACTTAATTTGTTGTTTGGTTGGCAAGTTCGGGATAAGTTATCCCGGGATTAATAATTAGTACCGGGATAAGTTATCCCT

Pain1-Sb2 CACTTAATTTGTTGTTTGGTTGGCAAGTTCGGGATAAGTTATCCCGGGATTAATAATTAGTACCGGGATAAGTTATCCCT

Pain1-Db2 CACTTAATTTGTTGTTTGGTTGGCAAGTTCGGGATAACTTATCCCGGGATTAATAATTAGTACCGGGATAAGTTATCCCT

Pain1-Sb3 CACTTAATTTGTTGTTTGGTTGGCAAGTTCGGGATAACTTATCCCGGGATTAATAATTAGTACCGGGATAAGTTATCCCT

Pain1-Da CACTTAATTTGTTGTTTGGTTGGCAAGTTCGGGATAACTTATCCCGGGATTAATAATTAGTACCGGGATAAGTTATCCCT

Pain1-Sa CACTTAATTTGTTGTTTGGTTGGCAAGTTCGGGATAACTTATCCCGGGATTAATAATTAGTACCGGGATAAGTTATCCCT

Pain1-Dc CACTTAATTTGTTGTTTGGTTGGCAAGTTCGGGATAACTTATCCCGGGATTAATAATTAGTACCGGGATAAGTTATCCCT

Pain1-Sb1 CACTTAATTTGTTGTTTGGTTGGCAAGTTCGGGATAACTTATCCCGAGATTAATAATTAGTACCGGGATAAGTTATCCCT

Pain1-Db1 CCCCTTGGG-GATATAGTAATCCCG---------------GGATAAAATAGGTAAATGACAAAAATGTCTTTTTCAACCC

Pain1-Sb2 CCCCTTGGG-GATATAGTAATCCCG---------------GGATAAAATAGGTAAATGACAAAAATGTCTTTTTCAACCC

Pain1-Db2 CCCCTTGGATGGTATAGTAATCCCGAGATAACTTATTCCGGGATAAAATAGGTAAATGACAAAAATGTCTCTTTCAACCC

Pain1-Sb3 CCCCTTGGATGGTATAGTAATCCCGAGATAACTTATCCCGGGATAAAATAGGTAAATGACAAAAATGTCTCTTTCAACCC

Pain1-Da CCCCTTGGATGGTATAGTAATCCCGAGATAACTTATCCCGGGATAAAATAGGTAAATGACAAAAATGTCTCTTTCAACCC

Pain1-Sa CCCCTTGGATGGTATAGTAATCCCGAGATAACTTATCCCGGGATAAAATAGGTAAATGACAAAAATGTCTCTTTCAACCC

Pain1-Dc CCCCTTGGATGGTATAGTAATCCCGAGATAACTTATTCCGGGATAAAATAGGTAAATGACAAAAATGTCTCTTTCAACCC

Pain1-Sb1 CCCCTTGGGTGGTATAGTAATCCCGAGATAACTTATCCCGGGATAAAATAGGTAAATGACAAAAATGTCTCTTTTAACCC

Pain1-Db1 TTTTGTTACATTACTTTTTACATTCATGAAAGACATTTTTATAAACAAATAAATTGTTCTTAAAATTTATTATTTTGAAT

Pain1-Sb2 TTTTGTTACATTACTTTTTACATTCATGAAAGACATTTTTATAAACAAATAAATTGTTCTTAAAATTTATTATTTTGAAT

Pain1-Db2 TTTTGTTACATCACTTTTTACATTCATGAAAGACATTTATATAAACAAATAAATTGTTCTTAAAATTTATTATTTTGAAT

Pain1-Sb3 TTTTGT-ACATCACTTTTTACATTCATGAAAGACATTTATATAAACAAATAAATTGTTCTTAAAATTTATTATTTTGAAT

Pain1-Da TTTTGTTACATCACTTTTTACATTCATGAAAGACATTTATATAAACAAATAAATTGTTCTTAAAATTTATTATTTTGAAT

Pain1-Sa TTTTGTTACATCACTTTTTACATTCATGAAAGACATTTATATAAACAAATAAATTGTTCTTAAAATTTATTATTTTGAAT

Pain1-Dc TTTTGTTACATCACTTTTTACATTCATGAAAGACATTTATATAAACAAATAAATTGTTCTTAAAATTTATTATTTTGAAT

Pain1-Sb1 TTTTGTTACATCACTTTTTACATTCATGAAAAACATTTTTATAAACAAATAAATTGTTCTTAAAATTTATTATTTTGAAT

Pain1-Db1 ACAACAAACCAAACACTCAATAAAAAATAATCTCATC---------------ATAAACTTATCTCATCATAACTTGAATT

Pain1-Sb2 ACAACAAACCAAACACTCAATAAAAAATAATCTCATC---------------ATAAACTTATCTCATCATAACTTGAATT

Pain1-Db2 ACAACAAACCAAACACTCAATAAAAAATAATCTCATCT--------------ATAAATTAATCTCATCATAACTTGAATT

Pain1-Sb3 ACAACAAACCAAACACTCAATAAAAAATAATCTCATC---------------ATAAACTTATCTCATCATAACTTGAATT

Pain1-Da ACAACAAACCAAACCCTCAATAAAAAATAATCTCATC---------------ATAA-CTTATTCCATCGTAACTTGAATT

Pain1-Sa ATAACAAATCAAACACTCAATAAAAAATAATCTCATC---------------ATAA-CTTATTCCATCATAACTTGAATT

Pain1-Dc ACAACAAACCAAACCCTCAATAAAAACTAATTTCATCGTAACTTATTCTATCATAA-TTAATCTCATCATAACTTGAATT

Pain1-Sb1 ACAACAAATCAAACACTCAATAAAAAATAATTTCATCATAACTTATCTAATCATAA-CTAATCTCATCATAACTTGAATT

Pain1-Db1 CAAACCAAACTAGCCTAAGGACCAAAATAAAGAATTTGCCAAACAGTAAGGATCATTTTGGTCATTTCTCTAATCCCAAG

Pain1-Sb2 CAAACCAAACTAGCCTAAGGACCAAAATAAAGAATTTGCCAAACAGTAAGGATCATTTTGGTCATTTCTCTAATCCCAAG

Pain1-Db2 CAAACCAAACTAGCCTAAGGACCAAAATAAAGAATTTGCCAAACAGTAAGGATCATTTTGGTCATTTCTCTAATCCTAAG

Pain1-Sb3 CAAACCAAACTAGCCTAAGGACCAAAATAAAGAATTTGCCAAACAGTAAGGATCATTTTGGTCATTTCTCTAATCCTAAG

Pain1-Da CAAACCAAACTAGC-TAAGGACCAAAATAAAGAATTTGCCAAACGGTAAGGATCATTTTGGTCATTTCTCTAATCCCAAG

Pain1-Sa CAAACCAAACTAGC-TAAGGACCAAAATAAAGAATTTGCCAAACGGTAAGGATCATTTTGGTCATTTCTCTAATCCCAAG

Pain1-Dc CAAACCAAACTAGCCTAAGGACCAAAATAAAGAATTTGCCAAACAGTAAGGATCATTTTGGTCATTTCTCTAATCCCAAG

Pain1-Sb1 CAAACCAAACTAGCCTAAGGACCAAAATAAAGAATTTGCCAAACAGTAAGGATCATTTTGGTCATTTCTCTAATCCCAAG

Pain1-Db1 TGTACCTCAAACTATACAAGCCTTTTCTCACTCAATTCAGTTGCCCCCTGTCATTTTCTGTGTTCATCACCTATATATAA

Pain1-Sb2 TGTACCTCAAACTATACAAGCCTTTTCTCACTCAATTCAGTTGCCCCCTGTCATTTTCTGTGTTCATCACCTATATATAA

Pain1-Db2 TGTACCTCAAACTATACAAGCCTTTTCTCACTCAATTCAGTTGCCCCCTGTCATTTTCTGTGTTCATCACCTATATATAA

Pain1-Sb3 TGTACCTCAAACTATACAAGCCTTTTCTCACTCAATTCAGTTGCCCCCTGTCATTTTCTGTGTTCATCACCTATATATAA

Pain1-Da TGTACCTCTAACTATACAAGCCTTTTCTCACTCAATTCAGTTGCCCCCTGTCATTTTCTGCGTTCATCACCTATATATAA

Pain1-Sa TGTACCTCAAACTATACAAGCCTTTTCTTACTCAATTCAGTTGCCCCCTGTCATTTTCTGCGTTCATCACCTATATATAA

Pain1-Dc TGTACCTCAAACTATACAAGCCCTTTCTCACTCAATTCAGTTACCCCCTGTCATTTTCTGTGTTCATCACCTATATATAA

Pain1-Sb1 TGTACCTCAAACTATACAAGCCTTTTCTCACTCAATTCAGTTGCCCCCTGTCATTTTCTGTGTTCATCCCCTATATATAA

Pain1-Db1 AGCAGTAGACTGGTAGCTTC-CCCAATCCTCTACCTTCCATTATGGCCACCCAGTACCATTCCAGTTATGACCTGGAAAA

Pain1-Sb2 AGCAGTAGACTGGTAGCTTC-CCCAATCCTCTACCTTCCATTATGGCCACCCAGTACCATTCCAGTTATGACCTGGAAAA

Pain1-Db2 AGCAGTAGACTGGTAGCTTC-CCCAATCCTCTACCTTCCATTATGGCCACCCAGTACCATTCCAGTTATGACCTGGAAAA

Pain1-Sb3 AGCAGTAGACTGGTAGCTTC-CCCAATCCTCTACCTTCCATTATGGCCACCCAGTACCATTCCAGTTATGACCTGGAAAA

Pain1-Da AGCAGTAGACTGGTAGCTTC-CCCAATCCTCTACCTTCCATTATGGCCACGCAGTACCATTCCAGTTATGACCCGGAAAA

Pain1-Sa AGCAGTAGACTGGTAGCTTC-CCCAATCCTCTACCTTCCATTATGGCCACGCAGTACCATTCCAGTTATGACCCGGAAAA

Pain1-Dc AGCAGTAGACTGGTAGCTTC-CCCAATCCTCTACCTTCCATTATGGCCACCCAGTACCATTCCAGTTATGACCCGGAAAA

Pain1-Sb1 AGCCGTAGACTAGTTGCTTCTCCCATTCCTCTATCTTCCATTATGGCCACCCAGTACCACTCAAGTTATGACCCGGAAAA

Pain1-Db1 CTCCGCCTCCCATTACACATTCCTCCCGGATCAACCCGATTCCGGCCACCGGAAGTCCCTTAAAATCATCTCCGGCATTT

Pain1-Sb2 CTCCGCCTCCCATTACACATTCCTCCCGGATCAACCCGATTCCGGCCACCGGAAGTCCCTTAAAATCATCTCCGGCATTT

Pain1-Db2 CTCCGCCTCCCATTACACATTCCTCCCGGATCAACCTGATTCCGGCCACCGGAAGTCCCTTAAAATCATCTCCGGCATTT

Pain1-Sb3 CTCCGCCTCCCATTACACATTCCTCCCGGATCAACCTGATTCCGGCCACCGGAAGTCCCTTAAAATCATCTCCGGCATTT

Pain1-Da CTCCGCCTCCCATTACACATTCCTCCCGGATCAACCCGATTCCGGCCACCGGAAGTCCCTTAAAATCATCTCCGGCATTT

Pain1-Sa CTCCGCCTCCCATTACACATTCCTCCCGGATCAACCCGATTCCGGCCACCGGAAGTCCCTTAAAATCATCTCCGGCATTT

Pain1-Dc CTCCGCCTCCCATTACACATTCCTCCCGGATCAACCCGATTCCGGCCACCGGAAGTCCCTTAAAATCATCTCCGGCATTT

Pain1-Sb1 CTCCGCCTCCCATTACACATTCCTCCCGGATCAACACGATTCCGGCCACCGGAAATCCCTTAAAATCATCTCCGGCATTT

Pain1-Db1 TCCTCTCCTCTTTCCTTTTGCTTTCTGTAGCCTTCTTTCCGATCCTCAACAACCAATCACCGGACTTGCAGAGTAACTCC

Pain1-Sb2 TCCTCTCCTCTTTCCTTTTGCTTTCTGTAGCCTTCTTTCCGATCCTCAACAACCAATCACCGGACTTGCAGAGTAACTCC

Pain1-Db2 TCCTCTCCTCTTTCCTTTTGCTTTCTGTAGCCTTCTTTCCGATCCTCAACAACCAATCACCGGACTTGCAGAGTAACTCC

Pain1-Sb3 TCCTCTCCTCTTTCCTTTTGCTTTCTGTAGCCTTCTTTCCGATCCTCAACAACCAATCACCGGACTTGCAGAGTAACTCC

Pain1-Da TCCTCTCCTCTTTCCTTTTGCTTTCTGTAGCCTTCTTTCCGATCCTCAACAACCAGTCACCGGACTTGCAGAGTAACTCC

Pain1-Sa TCCTCTCCTCTTTCCTTTTGCTTTCTGTAGCCTTCTTTCCGATCCTCAACAACCAGTCACCGGACTTGCAGAGTAACTCC

Pain1-Dc TCCTCTCCTCTTTCCTTTTGCTTTCTGTAGCCTTCTTTCCGATCCTCAACAACCAGTCACCGGACTTGCAGAGTAACTCC

Pain1-Sb1 TCCTCTCCTCTCTCCTTTTGCTTTCTTTAGTCTTCTTTCCGATCCTCAACAACCAGTCACCGGACTTGAAAAGTAACGCC

Pain1-Db1 CGTTCGCCGGCGCCGCCGTCAAGAGGTGTTTCTCAGGGAGTCTCCGATAAGACTTTTCGAGATGTCGTCAATGCTAGTCA

Pain1-Sb2 CGTTCGCCGGCGCCGCCGTCAAGAGGTGTTTCTCAGGGAGTCTCCGATAAGACTTTTCGAGATGTCGTCAATGCTAGTCA

Pain1-Db2 CGTTCGCCGGCGCCGCCGTCAAGAGGTGTTTCTCAGGGAGTCTCCGATAAGACTTTTCGAGATGTCGTCAATGCTAGTCA

Pain1-Sb3 CGTTCGCCGGCGCCGCCGTCAAGAGGTGTTTCTCAGGGAGTCTCCGATAAGACTTTTCGAGATGTCGTCAATGCTAGTCA

Pain1-Da CGTTCGCCGGCGCCGCCGTCAAGAGGTGTTTCTCAGGGAGTCTCCGATAAGACTTTTCGAGATGTCGTCAATGCTAGTCA

Pain1-Sa CGTTCGCCGGCGCCACCGTCAAGAGGTGTTTCTCAGGGAGTCTCCGATAAGACTTTTCGAGATGTCGTCAATGCTAGTCA

Pain1-Dc CGTTCGCCGGCGCCGCCGTCAAGAGGTGTTTCTCAGGGAGTCTCCGATAAGACTTTTCGAGATGTCGTCAATGCTAGTCA

Pain1-Sb1 CGTTCGCCGGCGCCGCCGTCAAGAGGTGTTTCTCAGGGAGTCTCCGATAAGACTTTTCGAGATGTCGTCAATGCTAGTCA

Pain1-Db1 CGTTTCTTATGCGTGGTCCAATGCTATGCTTAGCTGGCAAAGAACTGCTTACCATTTTCAACCAATCGAATTCCCGCGGC

Pain1-Sb2 CGTTTCTTATGCGTGGTCCAATGCTATGCTTAGCTGGCAAAGAACTGCTTACCATTTTCAACCAATCGAATTCCCGCGGC

Pain1-Db2 CGTTTCTTATGCGTGGTCCAATGCTATGCTTAGCTGGCAAAGAACTGCTTACCATTTTCAACCAATCGAATTCCCGCGGC

Pain1-Sb3 CGTTTCTTATGCGTGGTCCAATGCTATGCTTAGCTGGCAAAGAACTGCTTACCATTTTCAACCAATCGAATTCCCGCGGC

Pain1-Da CATTTCTTATGCGTGGTCCAATGCTATGCTTAGCTGGCAAAGAACTGCTTACCATTTTCAACCAATCGAATTCCCGCGGC

Pain1-Sa CGTTTCTTATGCGTGGTCCAATGCTATGCTTAGCTGGCAAAGAACTGCTTACCATTTTCAACCAATCGAATTCCCGCGGC

Pain1-Dc CGTTTCTTATGCGTGGTCCAATGCTATGCTTAGCTGGCAAAGAACTGCTTACCATTTTCAACCAATCGAATTCCCGCGGC

Pain1-Sb1 CGTTTCTTATGCGTGGTCCAATGCTATGCTTAGCTGGCAAAGAACTGCTTACCATTTTCAACCAATCGAATTCCCGCGGC
