# Supplementary material for: Validation of candidate gene markers for marker-assisted selection of potato cultivars with improved tuber quality
Source: Theor Appl Genet. 2013 Jan 9;126(4):1039–52. doi: 10.1007/s00122-012-2035-z (PMC3607734; doi:10.1007/s00122-012-2035-z)
Supplement: Supplementary file 2 — Supplementary material 2 (DOCX 15 kb) [file 122_2012_2035_MOESM2_ESM.docx]

**Supplementary Table 1**: Significant effects of single markers and marker combinations on tuber quality traits in 2009 and 2010 in 76 BNC clones.

| Marker  (No of genotypes having the marker) | CQA-09  p-value ^a^ | CQA-10  p-value ^a^ | CQS7-09  p-value ^a^ | CQS7-10  p-value ^a^ | CQS5-09  p-value ^a^ | CQS5-10  p-value ^a^ | TSC-09  p-value ^b^ | TSC-10  p-value ^b^ |
| --- | --- | --- | --- | --- | --- | --- | --- | --- |
| GP171-a (13) | ns | ns | ns | ns | 0.021↓ | ns | ns | ns |
| StpL-3e (47) | ns | ns | ns | ns | ns | ns | 0.016↑ | 0.012↑ |
| Stp23-8b (35) | ns | ns | ns | ns | 0.026↑ | ns | < 0 .001↑ | < 0 .001↑ |
| Pain1-9a (39) | ns | ns | ns | ns | ns | ns | 0.004↑ | 0.004↑ |
| Pain1-8c (28) | ns | ns | ns | 0.002↑ | ns | ns | ns | ns |
| Pain1_prom_-d/e (28) | ns | ns | ns | 0.004↑ | ns | ns | ns | ns |
| AGPsS-9a (18) | 0.008↑ | ns | 0.032↑ | ns | ns | 0.045↑ | ns | ns |
| Pain1-8c /AGPsS-9a | 0.047 | ns | ns | 0.002 | ns | ns | ns | ns |
| Stp23-8b/AGPsS-9a | 0.049 | ns | ns | ns | 0.038 | ns | < 0 .001 | 0 .001 |
| Stp23-8b/Pain1-8c | ns | ns | 0.027 | 0.024 | 0.009 | ns | < 0 .001 | < 0 .001 |
| StpL-3e/AGPsS-9a | ns | ns | ns | ns | ns | ns | ns | ns |
| GP171-a/AGPsS-9a | ns | ns | 0.042 | ns | 0.016 | ns | ns | ns |
| GP171-a/Stp23-8b | ns | ns | ns | ns | 0.045 | ns | < 0 .001 | < 0 .001 |
| GP171-a/Pain1-8c | ns | ns | ns | 0.020 | 0.043 | ns | ns | 0.026 |
| Stp23-8b/StpL-3e | ns | ns | ns | ns | 0.013 | ns | < 0 .001 | < 0 .001 |
| Pain1-8c /AGPsS-9a/ Stp23-8b | ns | ns | ns | 0.027 | 0.025 | ns | 0.001 | 0 .001 |
| GP171/ AGPsS-9a/ Stp23-8b | ns | ns | ns | ns | 0.030 | ns | 0.001 | 0.003 |
| GP171-a/ Pain1-8c/ Stp23-8b | ns | ns | 0.040 | ns | 0.010 | ns | 0.001 | < 0 .001 |

^a^ Mann-Whitney-U test or Kruskall-Wallis test, ns: not significant

^b^ t-test and ANOVA, ns: not significant

^c^ Direction of effect : ↑ the marker has a positive effect on the trait (on average higher tuber starch content and lighter chip color), ↓ the marker has a negative effect on the trait (on average lower tuber starch content and darker chip color)
